# Supplementary figures and images for: Unraveling the molecular pathobiology of vocal fold systemic dehydration using an in vivo rabbit model
Source: PLoS One. 2020 Jul 31;15(7):e0236348. doi: 10.1371/journal.pone.0236348 (PMC7394397; doi:10.1371/journal.pone.0236348)

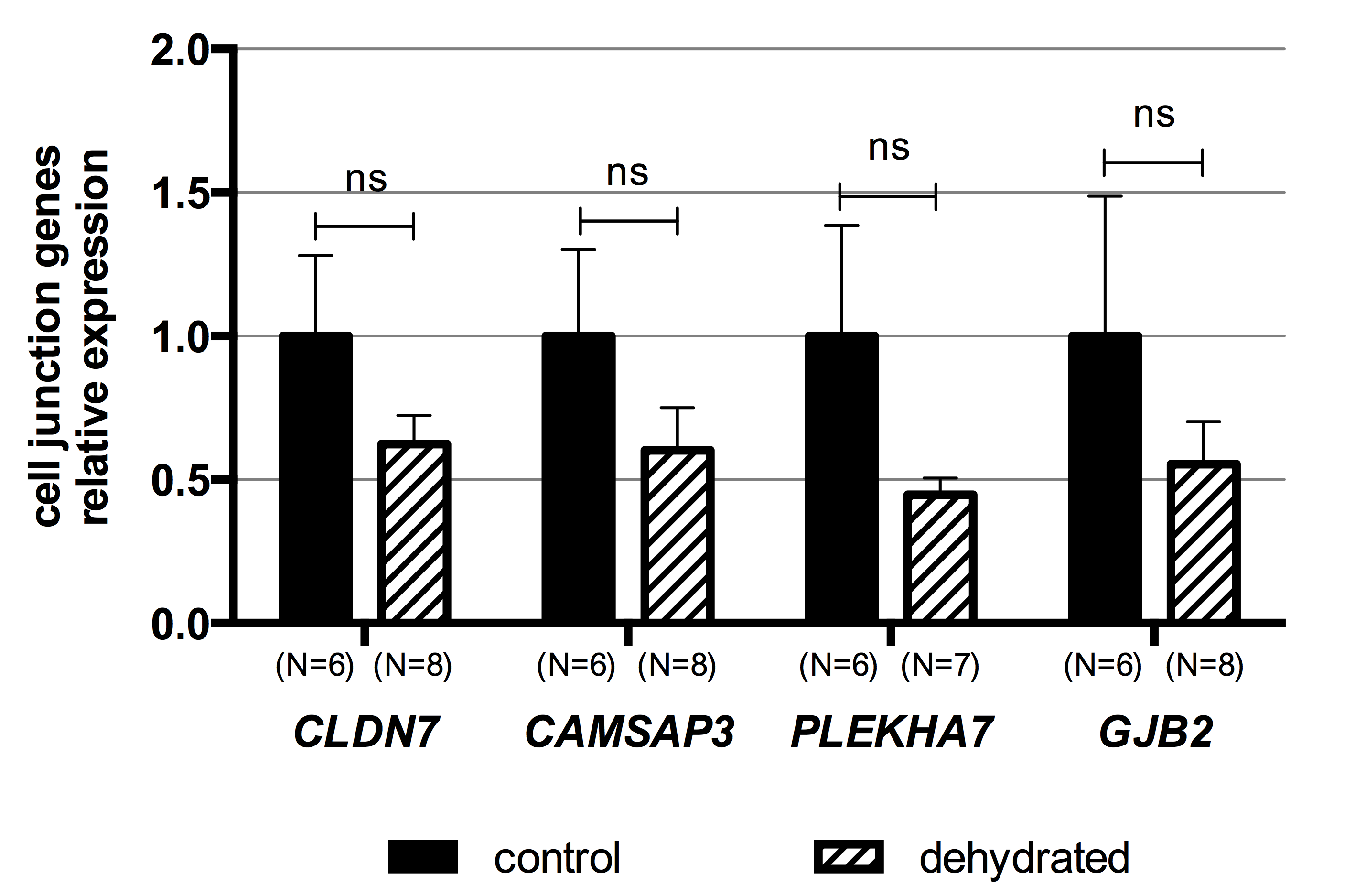

Supplement: S1 Fig — Genes: claudin 7 (CLDN7), calmodulin regulated spectrin associated protein family member 3 (CAMSAP3), pleckstrin homology domain containing A7 (PLEKHA7), and gap junction protein beta 2 (GJB2). The gene expression levels of the control group were set to 1, and relative expression levels were calculated relative to the HPRT1 gene using the method 2-ΔΔCt. Bars show mean ± SEM. ns: non-significant. (TIFF) [file pone.0236348.s001.tiff]
