# Supplementary material for: Unraveling the molecular pathobiology of vocal fold systemic dehydration using an in vivo rabbit model
Source: PLoS One. 2020 Jul 31;15(7):e0236348. doi: 10.1371/journal.pone.0236348 (PMC7394397; doi:10.1371/journal.pone.0236348)
Supplement: S1 Table — (PDF) [file pone.0236348.s002.pdf]

S1 Table. Summary of pairwise analysis of hematologic analytes as markers of systemic dehydration.

	Blood analyte	% change in the blood (mean ± SEM) Control (N=6)	Dehydrated (N=8)	Mann-Whitney p-value*
	PCV	-0.09 ± 1.26	15.64 ± 1.74	0.0003
	TPP	-0.27 ± 1.66	16.27 ± 1.92	0.0003
**iSTAT Chem8+**	Creatinine	7.01 ± 3.22	49.44 ± 7.78	0.0013
	BUN	27.27 ± 4.45	64.33 ± 8.46	0.0023
	Glucose	1.15 ± 4.46	32.23 ± 10.02	0.0593
	Sodium	1.08 ± 0.75	-1.32 ± 0.50	0.0273
	Chloride	3.00 ± 1.54	-4.82 ± 1.53	0.008
	Potassium	-4.50 ± 2.36	-11.60 ± 2.79	0.2393; ns
	Total CO_2	3.42 ± 9.34	15.09 ± 15.57	0.9760; ns
	iCa	-0.21 ± 3.76	-6.86 ± 2.00	0.2671; ns
	Anion gap	-4.03 ± 10.89	3.58 ± 4.99	0.2684; ns
	Hematocrit	-2.05 ± 1.49	14.77 ± 1.52	0.0003
	Hemoglobin	-2.05 ± 1.45	14.73 ± 1.53	0.0003

PCV: packed cell volume; TPP: total plasma protein; BUN: blood urea nitrogen; iCa: ionized calcium
*Exact p-value: differences between control and dehydrated group are statistically significant when $p \leq 0.05$.
ns: non-significant
