# Supplementary material for: Unraveling the molecular pathobiology of vocal fold systemic dehydration using an in vivo rabbit model
Source: PLoS One. 2020 Jul 31;15(7):e0236348. doi: 10.1371/journal.pone.0236348 (PMC7394397; doi:10.1371/journal.pone.0236348)
Supplement: S2 Table — Columns description: Sample ID: sample names in the study; Total Read Pairs: number of total read pairs; Quality Control Read Pairs: number of read pairs after quality control, and % of reads that passed quality control; Total Mapped Read Pairs: number of reads mapped to the genome, and % of reads mapped to the genome; Uniquely Mapped Read Pairs: number of reads uniquely mapped to the genome, and % of reads uniquely mapped to the genome; Read pairs went into genes: number of reads in genic regions, and % of reads mapped to the genes. (PDF) [file pone.0236348.s003.pdf]

S2 Table. Statistics of quality and mapping of the reads generated by RNA-Seq of vocal fold samples.

Sample ID	Total Read Pairs	Quality Control Read Pairs		Total Mapped Read Pairs (based on quality control read pairs)		Uniquely Mapped Read Pairs (based on quality control read pairs)		Read pairs went into genes (based on total mapped read pairs)	
		Number	%	Number	%	Number	%	Number	%
Rabbit 240 (12) control	66143667	66100178	99.93	58522126	88.54	53663767	81.19	42496048	72.62
Rabbit 869 (14) control	107755671	107687037	99.94	95434601	88.62	79169746	73.52	62129681	65.10
Rabbit 964 (17) control	87408450	87352548	99.94	79773825	91.32	72736548	83.27	57458318	72.03
Rabbit 921 (21) control	125274050	125183809	99.93	111966040	89.44	89676470	71.64	69670859	62.22
Rabbit 239 (13) dehydrated	84204744	84139472	99.92	74056563	88.02	69002208	82.01	54560066	73.67
Rabbit 963 (15) dehydrated	75775868	75723442	99.93	67942599	89.72	61779793	81.59	49590267	72.99
Rabbit 875 (16) dehydrated	99267239	99197774	99.93	90013349	90.74	81684791	82.35	64672009	71.85
Rabbit 876 (18) dehydrated	64259345	64213401	99.93	57827885	90.06	49691359	77.38	39039165	67.51
Rabbit 919 (19) dehydrated	78974509	78923352	99.94	71586258	90.70	58774914	74.47	45847047	64.04
Rabbit 922 (22) dehydrated	117741119	117634905	99.91	105735235	89.88	85466835	72.65	66666313	63.05
