# Supplementary material for: Unraveling the molecular pathobiology of vocal fold systemic dehydration using an in vivo rabbit model
Source: PLoS One. 2020 Jul 31;15(7):e0236348. doi: 10.1371/journal.pone.0236348 (PMC7394397; doi:10.1371/journal.pone.0236348)
Supplement: S3 Table — Only differentially expressed genes (q-value ≤ 0.05) are shown in the table. Columns description: Ensembl gene ID: describes the Ensembl gene identity; Sample_1: control; Sample_2: dehydrated; Value_1: FPKM of the gene in the control group; Value_2: FPKM of the gene in the dehydrated group; log2fold_change: describes log to base 2 value of fold change (Value_2/Value_1); q-value: The FDR-adjusted p-value of the test statistic; Gene symbol: official gene symbol; Description: annotation from Biomart. Note: All the log2 fold change values describe the fold change in the dehydrated group compared to control. Therefore, negative values represent downregulation, and positive values represent the upregulation of a given gene in the dehydrated group. (PDF) [file pone.0236348.s004.pdf]

S3 Table. Differential expression analysis between control and dehydrated vocal fold samples using Cufflinks.

Ensembl gene ID	Sample_1	Sample_2	Value_1	Value_2	log2fold_ change	q-value	Gene symbol	Description
ENSOCUG00000000197	Control	Dehydrated	26.1764	62.0782	1.24582	0.00669	*HK2*	hexokinase 2 [Source:NCBI gene;Acc:100338214]
ENSOCUG00000000737	Control	Dehydrated	3.34009	79.3614	4.57048	0.00669	*NR4A3*	nuclear receptor subfamily 4 group A member 3 [Source:NCBI gene;Acc:100344785]
ENSOCUG00000000786	Control	Dehydrated	25.9838	11.9905	-1.11572	0.00669	*EPHA2*	EPH receptor A2 [Source:HGNC Symbol;Acc:HGNC:3386]
ENSOCUG00000001054	Control	Dehydrated	27.8884	13.1614	-1.08335	0.00669	*GLTP*	glycolipid transfer protein [Source:NCBI gene;Acc:100347859]
ENSOCUG00000001114	Control	Dehydrated	21.3102	2.67637	-2.99319	0.00669	*TGM3*	transglutaminase 3 [Source:NCBI gene;Acc:100351990]
ENSOCUG00000001230	Control	Dehydrated	13.3655	5.13995	-1.37869	0.00669	*XDH*	xanthine dehydrogenase [Source:NCBI gene;Acc:100144327]
ENSOCUG00000001484	Control	Dehydrated	0	0.78568	#NUM!	0.00669	*IMPG1*	interphotoreceptor matrix proteoglycan 1 [Source:HGNC Symbol;Acc:HGNC:6055]
ENSOCUG00000001869	Control	Dehydrated	119.62	42.3207	-1.49903	0.00669		unknown
ENSOCUG00000002341	Control	Dehydrated	42.3336	20.2418	-1.06447	0.00669	*CAPG*	capping actin protein, gelsolin like [Source:NCBI gene;Acc:100347131]
ENSOCUG00000002371	Control	Dehydrated	13.8412	4.21578	-1.7151	0.00669	*SYTL1*	synaptotagmin like 1 [Source:NCBI gene;Acc:100343322]
ENSOCUG00000002424	Control	Dehydrated	240.562	63.8756	-1.91307	0.00669		Serum amyloid A protein
ENSOCUG00000002745	Control	Dehydrated	546.658	167.601	-1.70561	0.00669	*S100A14*	S100 calcium binding protein A14 [Source:NCBI gene;Acc:100345947]
ENSOCUG00000002944	Control	Dehydrated	6.54943	1.49599	-2.13027	0.00669	*SLC34A2*	solute carrier family 34 member 2 [Source:NCBI gene;Acc:100345994]
ENSOCUG00000003577	Control	Dehydrated	706.831	92.642	-2.93163	0.00669	*SCGB1D1*	Oryctolagus cuniculus lipophilin AL (LOC100008792), mRNA. [Source:RefSeq mRNA;Acc:NM_001082092]
ENSOCUG00000004161	Control	Dehydrated	144.818	58.7065	-1.30264	0.00669	*IL1RN*	interleukin 1 receptor antagonist [Source:NCBI gene;Acc:100009558]
ENSOCUG00000004625	Control	Dehydrated	212.308	52.6072	-2.01282	0.00669	*ORM1*	Oryctolagus cuniculus orosomucoid 1 (ORM1), mRNA. [Source:RefSeq mRNA;Acc:NM_001101695]
ENSOCUG00000004655	Control	Dehydrated	49.0482	21.6419	-1.18037	0.00669	*CYP2B4*	Oryctolagus cuniculus cytochrome P450, family 2, subfamily b, polypeptide 4 (CYP2B4), mRNA. [Source:RefSeq mRNA;Acc:NM_001170859]
ENSOCUG00000004918	Control	Dehydrated	166.211	78.4839	-1.08255	0.00669	*FAM129B*	family with sequence similarity 129 member B [Source:HGNC Symbol;Acc:HGNC:25282]
ENSOCUG00000005107	Control	Dehydrated	607.98	231.176	-1.39503	0.00669	*HOPX*	HOP homeobox [Source:NCBI gene;Acc:100037714]
ENSOCUG00000005202	Control	Dehydrated	1391.02	603.28	-1.20525	0.00669	*S100A11*	S100 calcium binding protein A11 [Source:NCBI gene;Acc:100008975]
ENSOCUG00000005417	Control	Dehydrated	53.7176	9.70867	-2.46805	0.00669	*TMPRSS11B*	transmembrane serine protease 11B [Source:HGNC Symbol;Acc:HGNC:25398]
ENSOCUG00000005634	Control	Dehydrated	103.252	42.3821	-1.28465	0.00669	*RAB25*	RAB25, member RAS oncogene family [Source:NCBI gene;Acc:100009059]
ENSOCUG00000006251	Control	Dehydrated	37.262	15.9761	-1.22178	0.00669	*EPHX2*	epoxide hydrolase 2 [Source:NCBI gene;Acc:100350230]
ENSOCUG00000006275	Control	Dehydrated	95.9433	11.9006	-3.01114	0.00669	*PIP*	prolactin induced protein [Source:NCBI gene;Acc:100328927]
ENSOCUG00000006280	Control	Dehydrated	55.2567	15.2969	-1.85291	0.00669	*CDSN*	corneodesmosin [Source:NCBI gene;Acc:100338321]
ENSOCUG00000006405	Control	Dehydrated	339.327	118.694	-1.51542	0.00669	*SFN*	stratifin [Source:NCBI gene;Acc:100358179]
ENSOCUG00000006494	Control	Dehydrated	51.0985	12.555	-2.02502	0.00669	*CNFN*	cornifelin [Source:NCBI gene;Acc:100357018]
ENSOCUG00000006833	Control	Dehydrated	0.27511	1.1773	2.09742	0.00669	*DNAH12*	dynein axonemal heavy chain 12 [Source:NCBI gene;Acc:100353849]
ENSOCUG00000007586	Control	Dehydrated	0.7856	2.89332	1.88085	0.00669	*SPAG17*	sperm associated antigen 17 [Source:NCBI gene;Acc:100353938]
ENSOCUG00000007730	Control	Dehydrated	30.5061	13.3434	-1.19297	0.00669	*LAD1*	ladinin 1 [Source:NCBI gene;Acc:100343996]
ENSOCUG00000007958	Control	Dehydrated	393.099	132.669	-1.56706	0.00669	*LY6G6C*	lymphocyte antigen 6 family member G6C [Source:NCBI gene;Acc:100351585]
ENSOCUG00000008632	Control	Dehydrated	497.168	74.9568	-2.7296	0.00669	*S100A9*	S100 calcium binding protein A9 [Source:NCBI gene;Acc:100008704]
ENSOCUG00000008689	Control	Dehydrated	129.389	48.6895	-1.41003	0.00669	*CEACAM1*	carcinoembryonic antigen related cell adhesion molecule 1 [Source:NCBI gene;Acc:100351299]
ENSOCUG00000008771	Control	Dehydrated	3.13206	0.85715	-1.8695	0.00669	*IL1A*	interleukin 1 alpha [Source:NCBI gene;Acc:100009250]
ENSOCUG00000008779	Control	Dehydrated	120.781	43.8849	-1.4606	0.00669	*SPINK5*	serine peptidase inhibitor, Kazal type 5 [Source:NCBI gene;Acc:100337963]
ENSOCUG00000008818	Control	Dehydrated	152.436	67.0454	-1.18499	0.00669	*CKB*	Oryctolagus cuniculus creatine kinase B (CKB), mRNA. [Source:RefSeq mRNA;Acc:NM_001082261]
ENSOCUG00000009148	Control	Dehydrated	93.7372	40.0405	-1.22716	0.00669	*SERPINB11*	serpin family B member 11 [Source:NCBI gene;Acc:100328646]
ENSOCUG00000009909	Control	Dehydrated	22.6676	8.19033	-1.46864	0.00669	*PHYHIP*	phytanoyl-CoA 2-hydroxylase interacting protein [Source:HGNC Symbol;Acc:HGNC:16865]
ENSOCUG00000010144	Control	Dehydrated	15.6273	4.7022	-1.73266	0.00669	*TTC22*	tetratricopeptide repeat domain 22 [Source:NCBI gene;Acc:100339658]
ENSOCUG00000010205	Control	Dehydrated	0.98022	4.77708	2.28495	0.00669	*DNAH5*	dynein axonemal heavy chain 5 [Source:HGNC Symbol;Acc:HGNC:2950]
ENSOCUG00000010425	Control	Dehydrated	79.5537	25.9732	-1.61491	0.00669	*FABP5*	fatty acid binding protein 5 [Source:NCBI gene;Acc:100345743]
ENSOCUG00000010458	Control	Dehydrated	34.4903	15.4461	-1.15895	0.00669	*NECTIN1*	nectin cell adhesion molecule 1 [Source:NCBI gene;Acc:100341399]
ENSOCUG00000010912	Control	Dehydrated	18.0226	1.48383	-3.60241	0.00669	*KRTDAP*	keratinocyte differentiation associated protein [Source:NCBI gene;Acc:103345425]
ENSOCUG00000010917	Control	Dehydrated	90.2615	24.1113	-1.9044	0.00669	*SBSN*	suprabasin [Source:NCBI gene;Acc:100346157]
ENSOCUG00000010998	Control	Dehydrated	45.8753	16.9615	-1.43546	0.00669	*IL36RN*	interleukin 36 receptor antagonist [Source:NCBI gene;Acc:100340988]
ENSOCUG00000011082	Control	Dehydrated	30.6903	11.963	-1.35921	0.00669	*EPHA1*	EPH receptor A1 [Source:NCBI gene;Acc:100338483]
ENSOCUG00000011658	Control	Dehydrated	135.065	39.8046	-1.76265	0.00669	*NCCRP1*	non-specific cytotoxic cell receptor protein 1 homolog (zebrafish) [Source:NCBI gene;Acc:100357987]
ENSOCUG00000012584	Control	Dehydrated	254.713	31.5589	-3.01275	0.00669	*CA6*	carbonic anhydrase 6 [Source:NCBI gene;Acc:100358016]
ENSOCUG00000012774	Control	Dehydrated	1666.9	500.219	-1.73654	0.00669	*KRT4*	keratin 4 [Source:NCBI gene;Acc:100348388]
ENSOCUG00000012899	Control	Dehydrated	21.178	7.61089	-1.47643	0.00669	*TMEM79*	transmembrane protein 79 [Source:NCBI gene;Acc:100338752]
ENSOCUG00000013020	Control	Dehydrated	6.77975	1.74256	-1.96002	0.00669	*ADGRF4*	adhesion G protein-coupled receptor F4 [Source:NCBI gene;Acc:100352009]

ENSOCUG00000013357	Control	Dehydrated	29.7291	11.4328	-1.37869	0.00669	*GRHL3*	grainyhead like transcription factor 3 [Source:NCBI gene;Acc:100356381]
ENSOCUG00000013564	Control	Dehydrated	35.9598	72.2798	1.00721	0.00669	*TRIM63*	tripartite motif containing 63 [Source:NCBI gene;Acc:100347164]
ENSOCUG00000013593	Control	Dehydrated	0.14013	2.74882	4.29401	0.00669	*BPI*	bactericidal/permeability-increasing protein [Source:NCBI gene;Acc:100009228]
ENSOCUG00000013647	Control	Dehydrated	7.16528	15.6833	1.13014	0.00669	*SORBS1*	sorbin and SH3 domain containing 1 [Source:HGNC Symbol;Acc:HGNC:14565]
ENSOCUG00000013779	Control	Dehydrated	2.12019	0.33712	-2.65288	0.00669	*FOXN1*	forkhead box N1 [Source:NCBI gene;Acc:100346912]
ENSOCUG00000013920	Control	Dehydrated	38.6763	12.8837	-1.58591	0.00669	*KRT80*	keratin 80 [Source:NCBI gene;Acc:100350733]
ENSOCUG00000014012	Control	Dehydrated	1.3603	0.11171	-3.60608	0.00669	*A2ML1*	alpha-2-macroglobulin like 1 [Source:NCBI gene;Acc:100347314]
ENSOCUG00000014227	Control	Dehydrated	12.7416	65.9219	2.37121	0.00669	*SEC14L3*	SEC14 like lipid binding 3 [Source:NCBI gene;Acc:100354633]
ENSOCUG00000014346	Control	Dehydrated	29.3871	11.9385	-1.29956	0.00669	*C1orf116*	chromosome 1 open reading frame 116 [Source:HGNC Symbol;Acc:HGNC:28667]
ENSOCUG00000014485	Control	Dehydrated	29.0944	12.9783	-1.16464	0.00669	*SPTBN2*	spectrin beta, non-erythrocytic 2 [Source:HGNC Symbol;Acc:HGNC:11276]
ENSOCUG00000014548	Control	Dehydrated	52.0048	21.5268	-1.27251	0.00669	*EVPL*	envoplakin [Source:HGNC Symbol;Acc:HGNC:3503]
ENSOCUG00000014668	Control	Dehydrated	6.84859	41.6114	2.6031	0.00669	*PPARGC1A*	PPARG coactivator 1 alpha [Source:NCBI gene;Acc:100349219]
ENSOCUG00000014998	Control	Dehydrated	157.569	54.0335	-1.54406	0.00669	*IL36A*	interleukin 36 alpha [Source:NCBI gene;Acc:100355520]
ENSOCUG00000015518	Control	Dehydrated	0	24.9291	#NUM!	0.00669	*CAMP*	cathelicidin antimicrobial peptide [Source:NCBI gene;Acc:100009142]
ENSOCUG00000015772	Control	Dehydrated	33.7438	10.271	-1.71605	0.00669	*CHI3L2*	chitinase-3-like protein 2 [Source:NCBI gene;Acc:100352595]
ENSOCUG00000016068	Control	Dehydrated	3.57609	12.4972	1.80515	0.00669	*SERPINE1*	serpin family E member 1 [Source:NCBI gene;Acc:100009588]
ENSOCUG00000016317	Control	Dehydrated	0.4669	1.72851	1.88834	0.00669	*CFAP44*	cilia and flagella associated protein 44 [Source:NCBI gene;Acc:100348161]
ENSOCUG00000016657	Control	Dehydrated	0	1.71401	#NUM!	0.00669	*EIF5A2*	eukaryotic translation initiation factor 5A2 [Source:NCBI gene;Acc:100355062]
ENSOCUG00000017034	Control	Dehydrated	6.26576	11.7981	0.912993	0.00669	*REV3L*	REV3 like, DNA directed polymerase zeta catalytic subunit [Source:NCBI gene;Acc:100359215]
ENSOCUG00000017247	Control	Dehydrated	4.91137	1.87862	-1.38645	0.00669	*CYP4F22*	cytochrome P450 family 4 subfamily F member 22 [Source:HGNC Symbol;Acc:HGNC:26820]
ENSOCUG00000017694	Control	Dehydrated	77.815	27.3311	-1.50951	0.00669	*KRT23*	keratin 23 [Source:NCBI gene;Acc:100358195]
ENSOCUG00000017717	Control	Dehydrated	30.1558	12.6487	-1.25344	0.00669	*GRHL1*	grainyhead like transcription factor 1 [Source:NCBI gene;Acc:100352813]
ENSOCUG00000021242	Control	Dehydrated	69.6692	28.8321	-1.27285	0.00669	*PKP1*	plakophilin 1 [Source:NCBI gene;Acc:100338080]
ENSOCUG00000021361	Control	Dehydrated	78.1115	33.6563	-1.21466	0.00669	*PDLIM2*	PDZ and LIM domain 2 [Source:NCBI gene;Acc:100353069]
ENSOCUG00000021508	Control	Dehydrated	13.7633	6.1062	-1.17248	0.00669	*INAVA*	innate immunity activator [Source:NCBI gene;Acc:100357252]
ENSOCUG00000022335	Control	Dehydrated	0	13.0699	#NUM!	0.00669		Oryctolagus cuniculus defensin NP-3a (LOC100009134), mRNA. [Source:RefSeq mRNA;Acc:NM_001082298]
ENSOCUG00000022365	Control	Dehydrated	0	0.30161	#NUM!	0.00669		unknown
ENSOCUG00000022549	Control	Dehydrated	36.101	15.6826	-1.20288	0.00669	*LOC100344499*	transmembrane protease serine 11G [Source:NCBI gene;Acc:100344499]
ENSOCUG00000022624	Control	Dehydrated	23.6539	10.0823	-1.23025	0.00669	*SLC26A9*	solute carrier family 26 member 9 [Source:NCBI gene;Acc:100346998]
ENSOCUG00000022645	Control	Dehydrated	0	0.67279	#NUM!	0.00669	*HPCAL4*	hippocalcin like 4 [Source:NCBI gene;Acc:100340413]
ENSOCUG00000022646	Control	Dehydrated	1584.74	213.426	-2.89244	0.00669	*S100A12*	S100 calcium binding protein A12 [Source:NCBI gene;Acc:100008703]
ENSOCUG00000023409	Control	Dehydrated	14.7718	6.15363	-1.26333	0.00669	*RAPGEFL1*	Rap guanine nucleotide exchange factor like 1 [Source:NCBI gene;Acc:100357164]
ENSOCUG00000023739	Control	Dehydrated	0.56353	3.86236	2.77692	0.00669	*AICDA*	activation induced cytidine deaminase [Source:HGNC Symbol;Acc:HGNC:13203]
ENSOCUG00000023878	Control	Dehydrated	2.48735	5.64879	1.18333	0.00669	*SYNE1*	spectrin repeat containing nuclear envelope protein 1 [Source:HGNC Symbol;Acc:HGNC:17089]
ENSOCUG00000024096	Control	Dehydrated	3190.28	1206.24	-1.40317	0.00669	*S100A6*	S100 calcium binding protein A6 [Source:NCBI gene;Acc:100348755]
ENSOCUG00000024745	Control	Dehydrated	15.3644	4.16122	-1.88451	0.00669	*TMPRSS11D*	transmembrane serine protease 11D [Source:HGNC Symbol;Acc:HGNC:24059]
ENSOCUG00000025291	Control	Dehydrated	26.5049	10.874	-1.28537	0.00669	*ST6GALNAC1*	ST6 N-acetylgalactosaminide alpha-2,6-sialyltransferase 1 [Source:NCBI gene;Acc:100357022]
ENSOCUG00000025736	Control	Dehydrated	28.0558	9.22162	-1.60521	0.00669	*APOBEC1*	apolipoprotein B mRNA editing enzyme catalytic subunit 1 [Source:NCBI gene;Acc:100009191]
ENSOCUG00000025810	Control	Dehydrated	9.49417	1.13238	-3.06768	0.00669	*TGM7*	transglutaminase 7 [Source:HGNC Symbol;Acc:HGNC:30790]
ENSOCUG00000025956	Control	Dehydrated	258.578	117.933	-1.13263	0.00669	*SDC1*	syndecan 1 [Source:NCBI gene;Acc:100338470]
ENSOCUG00000025990	Control	Dehydrated	44.6719	16.6673	-1.42235	0.00669	*PRSS27*	serine protease 27 [Source:HGNC Symbol;Acc:HGNC:15475]
ENSOCUG00000026264	Control	Dehydrated	76.459	23.7326	-1.68782	0.00669	*KLK13*	kallikrein related peptidase 13 [Source:HGNC Symbol;Acc:HGNC:6361]
ENSOCUG00000026334	Control	Dehydrated	17.4692	7.42184	-1.23496	0.00669	*GPNMB*	glycoprotein nmb [Source:NCBI gene;Acc:100350570]
ENSOCUG00000026555	Control	Dehydrated	20.2356	7.54576	-1.42316	0.00669		unknown
ENSOCUG00000027050	Control	Dehydrated	166.023	54.9554	-1.59505	0.00669	*LYPD3*	LY6/PLAUR domain containing 3 [Source:HGNC Symbol;Acc:HGNC:24880]
ENSOCUG00000027593	Control	Dehydrated	25.0858	4.54919	-2.46319	0.00669	*PSORS1C2*	psoriasis susceptibility 1 candidate 2 [Source:NCBI gene;Acc:100348579]
ENSOCUG00000027838	Control	Dehydrated	63.9199	20.1785	-1.66345	0.00669	*LOC100356345*	fatty acid desaturase 2-like protein FADS2P1 [Source:NCBI gene;Acc:100356345]
ENSOCUG00000028107	Control	Dehydrated	88.6987	34.5292	-1.3611	0.00669		unknown
ENSOCUG00000029153	Control	Dehydrated	11.1174	4.79523	-1.21314	0.00669	*CDH3*	cadherin 3 [Source:NCBI gene;Acc:100356285]
ENSOCUG00000029216	Control	Dehydrated	24.6297	6.42315	-1.93905	0.00669	*C17H15orf48*	chromosome 17 open reading frame, human C15orf48 [Source:NCBI gene;Acc:103351114]
ENSOCUG00000029244	Control	Dehydrated	59.6833	19.0435	-1.64803	0.00669	*KRT14*	keratin, type I cytoskeletal 14 [Source:NCBI gene;Acc:100344686]
ENSOCUG00000029278	Control	Dehydrated	202.155	69.1059	-1.54858	0.00669	*KRT78*	keratin 78 [Source:NCBI gene;Acc:100353913]
ENSOCUG00000029281	Control	Dehydrated	0.58329	2.94903	2.33796	0.00669	*LOC103351523*	C-C motif chemokine 15 [Source:NCBI gene;Acc:103351523]
ENSOCUG00000029569	Control	Dehydrated	18.9891	4.0783	-2.21913	0.00669	*KRT17*	keratin 17 [Source:NCBI gene;Acc:100344942]

ENSOCUG00000029663	Control	Dehydrated	2.13631	0.40444	-2.40112	0.00669	*KRT1*	keratin 1 [Source:NCBI gene;Acc:100353415]
ENSOCUG00000003200	Control	Dehydrated	4.36785	13.9145	1.67159	0.01171	*VGLL2*	vestigial like family member 2 [Source:NCBI gene;Acc:100347411]
ENSOCUG00000003467	Control	Dehydrated	539.436	210.897	-1.35492	0.01171	*PSCA*	prostate stem cell antigen [Source:NCBI gene;Acc:100344297]
ENSOCUG00000003544	Control	Dehydrated	140.491	46.738	-1.58781	0.01171	*MAL*	mal, T cell differentiation protein [Source:HGNC Symbol;Acc:HGNC:6817]
ENSOCUG00000004459	Control	Dehydrated	10.5338	25.4794	1.2743	0.01171	*KCNA1*	potassium voltage-gated channel subfamily A member 1 [Source:NCBI gene;Acc:100037722]
ENSOCUG00000004644	Control	Dehydrated	12.423	6.03092	-1.04257	0.01171	*CCDC120*	coiled-coil domain containing 120 [Source:HGNC Symbol;Acc:HGNC:28910]
ENSOCUG00000004852	Control	Dehydrated	213.277	94.2682	-1.17789	0.01171	*MALL*	mal, T cell differentiation protein like [Source:NCBI gene;Acc:100351475]
ENSOCUG00000006463	Control	Dehydrated	19.103	40.2003	1.0734	0.01171	*PRKAG2*	protein kinase AMP-activated non-catalytic subunit gamma 2 [Source:NCBI gene;Acc:100353180]
ENSOCUG00000008947	Control	Dehydrated	117.217	60.6217	-0.95127	0.01171	*MARCKSL1*	MARCKS like 1 [Source:HGNC Symbol;Acc:HGNC:7142]
ENSOCUG00000009682	Control	Dehydrated	6.45172	2.16235	-1.57708	0.01171	*GSDMA*	gasdermin A [Source:NCBI gene;Acc:100356912]
ENSOCUG00000010529	Control	Dehydrated	16.7409	2.75603	-2.60271	0.01171	*LOC100339150*	serpin B4 [Source:NCBI gene;Acc:100339150]
ENSOCUG00000011298	Control	Dehydrated	49.6029	20.6927	-1.2613	0.01171	*FAM83D*	family with sequence similarity 83 member D [Source:NCBI gene;Acc:100339633]
ENSOCUG00000011786	Control	Dehydrated	65.842	30.47	-1.11162	0.01171	*ECM1*	extracellular matrix protein 1 [Source:NCBI gene;Acc:100338579]
ENSOCUG00000012592	Control	Dehydrated	20.0058	40.4554	1.01591	0.01171	*ASB5*	ankyrin repeat and SOCS box containing 5 [Source:NCBI gene;Acc:100302410]
ENSOCUG00000028041	Control	Dehydrated	23.1684	9.72546	-1.25232	0.01171	*LOC100341802*	UDP-glucuronosyltransferase 2B31 [Source:NCBI gene;Acc:100341802]
ENSOCUG00000029190	Control	Dehydrated	34.2429	16.6478	-1.04048	0.01171	*PLAC8*	placenta specific 8 [Source:NCBI gene;Acc:100340512]
ENSOCUG00000004238	Control	Dehydrated	337.158	174.948	-0.9465	0.01599	*PERP*	PERP, TP53 apoptosis effector [Source:NCBI gene;Acc:100350501]
ENSOCUG00000008694	Control	Dehydrated	5.28062	12.9526	1.29446	0.01599	*DNAJC12*	DnaJ heat shock protein family (Hsp40) member C12 [Source:NCBI gene;Acc:100349695]
ENSOCUG00000011201	Control	Dehydrated	237.243	104.496	-1.18291	0.01599	*CYP4B1*	CYP4B1-like isozyme short form [Source:NCBI gene;Acc:100008805]
ENSOCUG00000011610	Control	Dehydrated	54.3724	104.481	0.942298	0.01599	*VEGFA*	vascular endothelial growth factor A [Source:NCBI gene;Acc:100008899]
ENSOCUG00000014187	Control	Dehydrated	0.7681	0.04936	-3.95996	0.01599	*MINAR1*	membrane integral NOTCH2 associated receptor 1 [Source:HGNC Symbol;Acc:HGNC:29172]
ENSOCUG00000014189	Control	Dehydrated	86.0971	43.1619	-0.99621	0.01599	*ANXA8*	annexin A8 [Source:NCBI gene;Acc:100008655]
ENSOCUG00000017373	Control	Dehydrated	13.3168	6.68049	-0.99522	0.01599	*PLEKHA7*	pleckstrin homology domain containing A7 [Source:HGNC Symbol;Acc:HGNC:27049]
ENSOCUG00000023796	Control	Dehydrated	35.4626	16.5947	-1.09558	0.01599	*LTF*	lactotransferrin [Source:NCBI gene;Acc:100348741]
ENSOCUG00000025113	Control	Dehydrated	8.58905	3.53785	-1.27963	0.01599	*MYCL*	MYCL proto-oncogene, bHLH transcription factor [Source:NCBI gene;Acc:100340331]
ENSOCUG00000025387	Control	Dehydrated	12.2822	24.4278	0.99196	0.01599		unknown
ENSOCUG00000026988	Control	Dehydrated	22.3859	9.74609	-1.1997	0.01599	*CHI3L1*	chitinase 3 like 1 [Source:NCBI gene;Acc:100338424]
ENSOCUG00000027480	Control	Dehydrated	0.63185	5.79091	3.19613	0.01599		unknown
ENSOCUG00000003071	Control	Dehydrated	15.0255	29.9181	0.99361	0.02025	*LPIN1*	lipin 1 [Source:NCBI gene;Acc:100346262]
ENSOCUG00000005775	Control	Dehydrated	3.11955	11.0062	1.8189	0.02025	*MYBL2*	MYB proto-oncogene like 2 [Source:NCBI gene;Acc:100346753]
ENSOCUG00000007989	Control	Dehydrated	6.11007	16.3998	1.42441	0.02025	*SPATA18*	spermatogenesis associated 18 [Source:NCBI gene;Acc:100357633]
ENSOCUG00000008688	Control	Dehydrated	2.28021	5.84446	1.35791	0.02025	*CFAP43*	cilia and flagella associated protein 43 [Source:NCBI gene;Acc:100348513]
ENSOCUG00000015371	Control	Dehydrated	25.1535	13.2784	-0.92168	0.02025	*SPINT1*	serine peptidase inhibitor, Kunitz type 1 [Source:NCBI gene;Acc:100345098]
ENSOCUG00000017421	Control	Dehydrated	218.642	110.162	-0.98895	0.02025	*KRT5*	keratin 5 [Source:NCBI gene;Acc:100352658]
ENSOCUG00000027112	Control	Dehydrated	2067.14	1091.06	-0.9219	0.02025	*LOC100358162*	60S ribosomal protein L28 [Source:NCBI gene;Acc:100358162]
ENSOCUG00000002632	Control	Dehydrated	155.43	77.7336	-0.99966	0.02378	*KRT7*	keratin 7 [Source:HGNC Symbol;Acc:HGNC:6445]
ENSOCUG00000004176	Control	Dehydrated	0.83187	0.07437	-3.48353	0.02378	*ADAM28*	ADAM metallopeptidase domain 28 [Source:HGNC Symbol;Acc:HGNC:206]
ENSOCUG00000004386	Control	Dehydrated	4.39804	10.5561	1.26315	0.02378	*NFAT5*	nuclear factor of activated T cells 5 [Source:NCBI gene;Acc:100343460]
ENSOCUG00000006498	Control	Dehydrated	11.508	5.79218	-0.99046	0.02378	*DSG1*	desmoglein 1 [Source:NCBI gene;Acc:100346111]
ENSOCUG00000012306	Control	Dehydrated	15.0428	7.69413	-0.96724	0.02378	*CDKN2B*	cyclin dependent kinase inhibitor 2B [Source:NCBI gene;Acc:100351874]
ENSOCUG00000013200	Control	Dehydrated	20.9362	8.48848	-1.30242	0.02378	*SDCBP2*	syndecan binding protein 2 [Source:NCBI gene;Acc:100349483]
ENSOCUG00000016033	Control	Dehydrated	5.68573	2.15655	-1.39862	0.02378	*WNT10A*	Wnt family member 10A [Source:NCBI gene;Acc:100352839]
ENSOCUG00000017120	Control	Dehydrated	10.0164	20.0477	1.00106	0.02378	*PDK4*	pyruvate dehydrogenase kinase 4 [Source:NCBI gene;Acc:100351580]
ENSOCUG00000021297	Control	Dehydrated	75.2817	36.755	-1.03436	0.02378	*TRIM29*	tripartite motif containing 29 [Source:NCBI gene;Acc:100340370]
ENSOCUG00000004691	Control	Dehydrated	7.5093	3.23464	-1.21508	0.02779	*PROM2*	prominin 2 [Source:NCBI gene;Acc:100358327]
ENSOCUG00000010057	Control	Dehydrated	0.81388	2.43064	1.57845	0.02779	*DNAH6*	dynein axonemal heavy chain 6 [Source:NCBI gene;Acc:100341245]
ENSOCUG00000011842	Control	Dehydrated	76.1288	27.1735	-1.48624	0.02779	*CRNN*	cornulin [Source:NCBI gene;Acc:100359146]
ENSOCUG00000023380	Control	Dehydrated	49.0972	22.7396	-1.11043	0.02779		unknown
ENSOCUG00000001241	Control	Dehydrated	43.6458	22.0711	-0.98368	0.03063	*MFSD4A*	major facilitator superfamily domain containing 4A [Source:NCBI gene;Acc:100348417]
ENSOCUG00000002128	Control	Dehydrated	14.5446	7.74087	-0.90992	0.03063	*ANKRD35*	ankyrin repeat domain 35 [Source:NCBI gene;Acc:100340763]
ENSOCUG00000002582	Control	Dehydrated	11.4364	5.52557	-1.04943	0.03063	*F2RL1*	F2R like trypsin receptor 1 [Source:HGNC Symbol;Acc:HGNC:3538]
ENSOCUG00000003276	Control	Dehydrated	9.93331	2.71933	-1.86902	0.03063	*PAX1*	paired box 1 [Source:HGNC Symbol;Acc:HGNC:8615]
ENSOCUG00000007002	Control	Dehydrated	169.508	87.0092	-0.96212	0.03063	*CLDN7*	claudin 7 [Source:NCBI gene;Acc:100338591]
ENSOCUG00000009566	Control	Dehydrated	478.365	123.203	-1.95708	0.03063	*S100A8*	S100 calcium binding protein A8 [Source:NCBI gene;Acc:100008985]

ENSOCUG00000013994	Control	Dehydrated	3.56826	0.35025	-3.34875	0.03063	*PLA2G4D*	phospholipase A2 group IVD [Source:NCBI gene;Acc:100340775]
ENSOCUG00000015184	Control	Dehydrated	0.30556	1.5619	2.35376	0.03063	*MYBL1*	MYB proto-oncogene like 1 [Source:NCBI gene;Acc:100345064]
ENSOCUG00000024304	Control	Dehydrated	6.07398	12.1436	0.999478	0.03063	*FNIP1*	folliculin interacting protein 1 [Source:NCBI gene;Acc:103347864]
ENSOCUG00000005112	Control	Dehydrated	59.8709	110.274	0.881168	0.03416	*FBXO32*	F-box protein 32 [Source:NCBI gene;Acc:100356177]
ENSOCUG00000007488	Control	Dehydrated	6.41429	2.71043	-1.24276	0.03416	*SUSD2*	sushi domain containing 2 [Source:NCBI gene;Acc:100358655]
ENSOCUG00000007849	Control	Dehydrated	0.69272	2.06189	1.57362	0.03416	*FHAD1*	forkhead associated phosphopeptide binding domain 1 [Source:HGNC Symbol;Acc:HGNC:29408]
ENSOCUG00000010156	Control	Dehydrated	31.0389	10.9566	-1.50227	0.03416	*GJB2*	gap junction protein beta 2 [Source:NCBI gene;Acc:100344821]
ENSOCUG00000003831	Control	Dehydrated	15.3373	7.97255	-0.94393	0.03687	*C4orf19*	chromosome 2 open reading frame, human C4orf19 [Source:NCBI gene;Acc:100351060]
ENSOCUG00000006774	Control	Dehydrated	0.43296	1.57569	1.86369	0.03687	*SPEF2*	sperm flagellar 2 [Source:NCBI gene;Acc:100353102]
ENSOCUG00000007545	Control	Dehydrated	10.9111	5.47011	-0.99615	0.03687	*EPHB6*	EPH receptor B6 [Source:HGNC Symbol;Acc:HGNC:3396]
ENSOCUG00000011323	Control	Dehydrated	6.10852	1.44467	-2.08008	0.03687	*DNASE1*	Oryctolagus cuniculus deoxyribonuclease 1 (DNASE1), mRNA. [Source:RefSeq mRNA;Acc:NM_001082740]
ENSOCUG00000012863	Control	Dehydrated	4.07123	10.6866	1.39226	0.03687	*CDHR3*	cadherin related family member 3 [Source:NCBI gene;Acc:100356106]
ENSOCUG00000017527	Control	Dehydrated	1.69531	4.64123	1.45296	0.03687	*LRRIQ1*	leucine rich repeats and IQ motif containing 1 [Source:NCBI gene;Acc:100339224]
ENSOCUG00000022231	Control	Dehydrated	384.473	212.87	-0.85291	0.03687	*EMP1*	epithelial membrane protein 1 [Source:NCBI gene;Acc:100009209]
ENSOCUG00000002730	Control	Dehydrated	21.4831	11.4274	-0.9107	0.0396	*LRRC1*	leucine rich repeat containing 1 [Source:NCBI gene;Acc:100355945]
ENSOCUG00000005298	Control	Dehydrated	31.9444	7.79483	-2.03497	0.0396	*KLK11*	kallikrein related peptidase 11 [Source:HGNC Symbol;Acc:HGNC:6359]
ENSOCUG00000013684	Control	Dehydrated	23.0734	7.80634	-1.56351	0.0396	*TMEM54*	transmembrane protein 54 [Source:NCBI gene;Acc:100340189]
ENSOCUG00000013917	Control	Dehydrated	1.2374	3.63828	1.55595	0.0396	*CFAP54*	cilia and flagella associated protein 54 [Source:NCBI gene;Acc:100350156]
ENSOCUG00000014245	Control	Dehydrated	15.3149	7.94758	-0.94635	0.0396	*METRNL*	meteorin like, glial cell differentiation regulator [Source:NCBI gene;Acc:100347364]
ENSOCUG00000016132	Control	Dehydrated	256.084	133.655	-0.9381	0.0396	*KRT19*	keratin, type I cytoskeletal 19 [Source:NCBI gene;Acc:100344434]
ENSOCUG00000002770	Control	Dehydrated	110.894	55.6321	-0.99519	0.04169	*FAM3D*	family with sequence similarity 3 member D [Source:NCBI gene;Acc:100355374]
ENSOCUG00000008654	Control	Dehydrated	11.401	2.52973	-2.1721	0.04169		unknown
ENSOCUG00000009646	Control	Dehydrated	6.15799	2.4338	-1.33925	0.04169	*SEC14L4*	SEC14-like protein 4 [Source:NCBI gene;Acc:100358382]
ENSOCUG00000011731	Control	Dehydrated	8.21451	2.36383	-1.79705	0.04169	*KRT10*	keratin 10 [Source:NCBI gene;Acc:100009029]
ENSOCUG00000012103	Control	Dehydrated	3.51804	1.85077	-0.92664	0.04169	*FAT2*	FAT atypical cadherin 2 [Source:NCBI gene;Acc:100347471]
ENSOCUG00000015114	Control	Dehydrated	18.1901	10.0369	-0.85785	0.04169	*ZNF710*	zinc finger protein 710 [Source:HGNC Symbol;Acc:HGNC:25352]
ENSOCUG00000015653	Control	Dehydrated	20.2581	6.32264	-1.6799	0.04169	*RNF39*	ring finger protein 39 [Source:NCBI gene;Acc:103349729]
ENSOCUG00000016131	Control	Dehydrated	17.8401	6.5465	-1.44633	0.04169	*IL20RB*	interleukin 20 receptor subunit beta [Source:HGNC Symbol;Acc:HGNC:6004]
ENSOCUG00000001374	Control	Dehydrated	1.64523	4.39039	1.41606	0.04476	*TSHR*	thyroid stimulating hormone receptor [Source:NCBI gene;Acc:100353965]
ENSOCUG00000016959	Control	Dehydrated	11.5078	6.36204	-0.85505	0.04476	*CRYBG2*	crystallin beta-gamma domain containing 2 [Source:NCBI gene;Acc:100337821]
ENSOCUG00000023000	Control	Dehydrated	6.13659	1.8297	-1.74583	0.04476		unknown
ENSOCUG00000004447	Control	Dehydrated	24.6607	13.5035	-0.86888	0.04774	*ALPL*	alkaline phosphatase, biomineralization associated [Source:HGNC Symbol;Acc:HGNC:438]
ENSOCUG00000011294	Control	Dehydrated	16.7623	7.72903	-1.11686	0.04774	*LOC103345257*	L-threonine 3-dehydrogenase, mitochondrial [Source:NCBI gene;Acc:103345257]
ENSOCUG00000017253	Control	Dehydrated	9.17812	4.69495	-0.96709	0.04774	*ARHGEF4*	Rho guanine nucleotide exchange factor 4 [Source:NCBI gene;Acc:100338395]
ENSOCUG00000000777	Control	Dehydrated	1.58753	0.39655	-2.0012	0.04888		unknown
ENSOCUG00000004067	Control	Dehydrated	6.70321	14.335	1.09662	0.04888	*CNST*	consortin, connexin sorting protein [Source:NCBI gene;Acc:100346480]
ENSOCUG00000006987	Control	Dehydrated	0.84786	2.97433	1.81067	0.04888	*TSGA10*	testis specific 10 [Source:NCBI gene;Acc:100348551]
ENSOCUG00000009400	Control	Dehydrated	0.62842	2.18092	1.79513	0.04888	*DNAI2*	dynein axonemal intermediate chain 2 [Source:NCBI gene;Acc:100356679]
ENSOCUG00000016105	Control	Dehydrated	10.2454	19.9724	0.96304	0.04888	*COLQ*	collagen like tail subunit of asymmetric acetylcholinesterase [Source:NCBI gene;Acc:100341444]
ENSOCUG00000016920	Control	Dehydrated	0.86682	2.96836	1.77586	0.04888	*NSUN7*	NOP2/Sun RNA methyltransferase family member 7 [Source:NCBI gene;Acc:100353331]
ENSOCUG00000016943	Control	Dehydrated	236.506	94.6519	-1.32117	0.04888	*FAM25A*	family with sequence similarity 25 member A [Source:NCBI gene;Acc:100348171]
ENSOCUG00000022921	Control	Dehydrated	12.3286	6.26588	-0.97642	0.04888	*CAMSAP3*	calmodulin regulated spectrin associated protein family member 3 [Source:HGNC Symbol;Acc:HGNC:29307]
ENSOCUG00000026763	Control	Dehydrated	903.962	387.431	-1.22232	0.04888		unknown
ENSOCUG00000027394	Control	Dehydrated	1614.42	766.483	-1.07469	0.04888		unknown
