# Supplementary material for: Unraveling the molecular pathobiology of vocal fold systemic dehydration using an in vivo rabbit model
Source: PLoS One. 2020 Jul 31;15(7):e0236348. doi: 10.1371/journal.pone.0236348 (PMC7394397; doi:10.1371/journal.pone.0236348)
Supplement: S4 Table — Columns description: Category: original database/resource of annotated terms (GOTERM_BP_FAT: biological process, GOTERM_CC_FAT: cellular component, GOTERM_MF_FAT: molecular function, KEGG_PATHWAY: KEGG pathway database); Term: enriched terms associated with gene list; Count: number of DE genes involved in the term; %: percentage of involved DE genes/total DEG genes mapped on DAVID analysis, e.g., 28*100/185 = 15.13%; p-value: modified Fisher exact p-value, the smaller, the more enriched; DE genes list: Ensembl gene IDs of genes involved in the term; List total: number of genes in the gene list mapped to any term in this ontology; Pop hits: number of genes with this term on the background list (genes in the genome); Pop total: number of genes on the background list mapped to any term in this ontology; Fold enrichment: defined as the ratio of the two proportions (% DEG involved in a term/% of background genes involved in the same term); FDR: False Discovery Rate. (PDF) [file pone.0236348.s005.pdf]

Table S4. Functional categories of differentially expressed (DE) genes in dehydrated vocal folds by GO_TERM FAT and KEGG pathway enrichment analysis.

Category	Term	Count	%	p-value	DE genes list	List total	Pop hits	Pop total	Fold enrichment	FDR
GOTERM_BP_FAT	GO:0009605~response to external stimulus	28	15.135	3.40E-04	ENSOCUG00000000786, ENSOCUG00000004447, ENSOCUG00000005107, ENSOCUG00000013593, ENSOCUG00000012306, ENSOCUG00000014668, ENSOCUG00000000737, ENSOCUG00000023739, ENSOCUG00000011610, ENSOCUG00000002424, ENSOCUG00000009566, ENSOCUG00000014245, ENSOCUG00000008779, ENSOCUG00000029281, ENSOCUG00000016068, ENSOCUG00000023796, ENSOCUG00000016131, ENSOCUG00000017120, ENSOCUG00000004459, ENSOCUG00000002745, ENSOCUG00000007002, ENSOCUG00000024304, ENSOCUG00000015518, ENSOCUG00000026988, ENSOCUG00000029190, ENSOCUG00000010998, ENSOCUG00000022335, ENSOCUG00000022646	118	1139	9777	2.03684469	0.587364
GOTERM_BP_FAT	GO:0006952~defense response	23	12.432	2.66E-05	ENSOCUG00000000786, ENSOCUG00000008771, ENSOCUG00000016068, ENSOCUG00000023796, ENSOCUG00000016131, ENSOCUG00000013593, ENSOCUG00000017120, ENSOCUG00000002745, ENSOCUG00000004625, ENSOCUG00000007545, ENSOCUG00000007002, ENSOCUG00000011786, ENSOCUG00000002424, ENSOCUG00000009566, ENSOCUG00000014998, ENSOCUG00000014245, ENSOCUG00000008779, ENSOCUG00000015518, ENSOCUG00000026988, ENSOCUG00000029190, ENSOCUG00000010998, ENSOCUG00000022335, ENSOCUG00000022646	118	705	9777	2.703101334	0.046197
GOTERM_BP_FAT	GO:0070887~cellular response to chemical stimulus	23	12.432	0.047393	ENSOCUG00000000786, ENSOCUG00000029281, ENSOCUG00000008771, ENSOCUG00000016033, ENSOCUG00000016068, ENSOCUG00000023796, ENSOCUG00000004447, ENSOCUG00000017120, ENSOCUG00000012306, ENSOCUG00000004459, ENSOCUG00000002745, ENSOCUG00000014668, ENSOCUG00000000737, ENSOCUG00000023739, ENSOCUG00000001230, ENSOCUG00000005112, ENSOCUG00000011610, ENSOCUG00000011786, ENSOCUG00000002424, ENSOCUG00000009566, ENSOCUG00000026988, ENSOCUG00000010998, ENSOCUG00000022646	118	1275	9777	1.494656032	56.91558
GOTERM_BP_FAT	GO:0008283~cell proliferation	21	11.351	0.015269	ENSOCUG00000000786, ENSOCUG00000011082, ENSOCUG00000008771, ENSOCUG00000016657, ENSOCUG00000005634, ENSOCUG00000023796, ENSOCUG00000016131, ENSOCUG00000012774, ENSOCUG00000006405, ENSOCUG00000012306, ENSOCUG00000004459, ENSOCUG00000007545, ENSOCUG00000000737, ENSOCUG00000001230, ENSOCUG00000029153, ENSOCUG00000008947, ENSOCUG00000011610, ENSOCUG00000011786, ENSOCUG00000013779, ENSOCUG00000029190, ENSOCUG00000011658	118	1004	9777	1.733042407	23.42039
GOTERM_BP_FAT	GO:0065009~regulation of molecular function	21	11.351	0.0525453	ENSOCUG00000000786, ENSOCUG00000011082, ENSOCUG00000016068, ENSOCUG00000004691, ENSOCUG00000023796, ENSOCUG00000004067, ENSOCUG00000005107, ENSOCUG00000006405, ENSOCUG00000013357, ENSOCUG00000012306, ENSOCUG00000014668, ENSOCUG00000007545, ENSOCUG00000001230, ENSOCUG00000029153, ENSOCUG00000022921, ENSOCUG00000024304, ENSOCUG00000011610, ENSOCUG00000011786, ENSOCUG00000009566, ENSOCUG00000008779, ENSOCUG00000026988	118	1146	9777	1.518302423	60.78299
GOTERM_BP_FAT	GO:0022610~biological adhesion	20	10.811	0.002409	ENSOCUG00000000786, ENSOCUG00000012863, ENSOCUG00000011082, ENSOCUG00000006498, ENSOCUG00000016068, ENSOCUG00000021242, ENSOCUG00000023796, ENSOCUG00000016131, ENSOCUG00000026334, ENSOCUG00000011842, ENSOCUG00000010458, ENSOCUG00000012103, ENSOCUG00000007545, ENSOCUG00000000737, ENSOCUG00000029153, ENSOCUG00000022921, ENSOCUG00000011610, ENSOCUG00000009566, ENSOCUG00000013779, ENSOCUG00000017373	118	787	9777	2.105614541	4.096448
GOTERM_BP_FAT	GO:0007155~cell adhesion	19	10.27	0.0052206	ENSOCUG00000000786, ENSOCUG00000012863, ENSOCUG00000011082, ENSOCUG00000006498, ENSOCUG00000016068, ENSOCUG00000021242, ENSOCUG00000016131, ENSOCUG00000026334, ENSOCUG00000011842, ENSOCUG00000010458, ENSOCUG00000012103, ENSOCUG00000007545, ENSOCUG00000000737, ENSOCUG00000029153, ENSOCUG00000022921, ENSOCUG00000011610, ENSOCUG00000009566, ENSOCUG00000013779, ENSOCUG00000017373	118	782	9777	2.013123672	8.677534
GOTERM_BP_FAT	GO:0042127~regulation of cell proliferation	19	10.27	0.0096304	ENSOCUG00000011082, ENSOCUG00000008771, ENSOCUG00000005202, ENSOCUG00000025736, ENSOCUG00000016657, ENSOCUG00000005634, ENSOCUG00000023796, ENSOCUG00000016131, ENSOCUG00000012774, ENSOCUG00000006405, ENSOCUG00000012306, ENSOCUG00000000737, ENSOCUG00000001230, ENSOCUG00000029153, ENSOCUG00000008947, ENSOCUG00000011610, ENSOCUG00000011786, ENSOCUG00000029190, ENSOCUG00000011658	118	831	9777	1.894419629	15.44941
GOTERM_BP_FAT	GO:0008219~cell death	19	10.27	0.0522758	ENSOCUG00000000786, ENSOCUG00000008771, ENSOCUG00000016068, ENSOCUG00000012899, ENSOCUG00000017120, ENSOCUG00000009682, ENSOCUG00000006405, ENSOCUG00000022231, ENSOCUG00000014668, ENSOCUG00000000737, ENSOCUG00000001230, ENSOCUG00000000197, ENSOCUG00000006275, ENSOCUG00000024304, ENSOCUG00000011610, ENSOCUG00000009566, ENSOCUG00000029190, ENSOCUG00000011323, ENSOCUG00000004918	118	1004	9777	1.567990749	60.58915
GOTERM_BP_FAT	GO:2000026~regulation of multicellular organismal development	19	10.27	0.0714852	ENSOCUG00000000786, ENSOCUG00000011082, ENSOCUG00000008771, ENSOCUG00000016033, ENSOCUG00000016068, ENSOCUG00000025113, ENSOCUG00000005107, ENSOCUG00000010458, ENSOCUG00000006405, ENSOCUG00000013357, ENSOCUG00000001230, ENSOCUG00000029153, ENSOCUG00000024304, ENSOCUG00000011610, ENSOCUG00000011786, ENSOCUG00000013779, ENSOCUG00000016105, ENSOCUG00000026988, ENSOCUG00000029569	118	1045	9777	1.506471495	72.36941
GOTERM_BP_FAT	GO:0042592~homeostatic process	18	9.7297	0.047595	ENSOCUG00000008771, ENSOCUG00000023796, ENSOCUG00000016131, ENSOCUG00000012899, ENSOCUG00000008818, ENSOCUG00000017120, ENSOCUG00000006405, ENSOCUG00000013357, ENSOCUG00000004459, ENSOCUG00000000737, ENSOCUG00000014668, ENSOCUG00000000197, ENSOCUG00000029153, ENSOCUG00000006275, ENSOCUG00000002944, ENSOCUG00000014245, ENSOCUG00000013779, ENSOCUG00000006251	118	923	9777	1.61582533	57.07377

GOTERM_BP_FAT	GO:0012501~programmed cell death	18	9.7297	0.0566515	ENSOCUG00000000786, ENSOCUG00000008771, ENSOCUG00000016068, ENSOCUG00000012899, ENSOCUG00000017120, ENSOCUG00000009682, ENSOCUG00000006405, ENSOCUG00000000737, ENSOCUG00000014668, ENSOCUG00000001230, ENSOCUG00000000197, ENSOCUG00000006275, ENSOCUG00000024304, ENSOCUG00000011610, ENSOCUG00000009566, ENSOCUG00000029190, ENSOCUG00000011323, ENSOCUG00000004918	118	944	9777	1.579880063	63.62847
GOTERM_BP_FAT	GO:0006955~immune response	17	9.1892	0.0042327	ENSOCUG00000029281, ENSOCUG00000008771, ENSOCUG00000023796, ENSOCUG00000016131, ENSOCUG00000013593, ENSOCUG00000002745, ENSOCUG00000007545, ENSOCUG00000000737, ENSOCUG00000023739, ENSOCUG00000007488, ENSOCUG00000009566, ENSOCUG00000011786, ENSOCUG00000014998, ENSOCUG00000008779, ENSOCUG00000015518, ENSOCUG00000010998, ENSOCUG00000022646	118	646	9777	2.180419269	7.091983
GOTERM_BP_FAT	GO:0060429~epithelium development	17	9.1892	0.0080018	ENSOCUG00000000786, ENSOCUG00000016033, ENSOCUG00000005634, ENSOCUG00000025113, ENSOCUG00000014548, ENSOCUG00000012899, ENSOCUG00000012774, ENSOCUG00000013357, ENSOCUG00000006405, ENSOCUG00000011731, ENSOCUG00000015371, ENSOCUG00000001230, ENSOCUG00000029153, ENSOCUG00000001114, ENSOCUG00000013779, ENSOCUG00000008779, ENSOCUG00000029569	118	691	9777	2.038423802	13.00557
GOTERM_BP_FAT	GO:0050790~regulation of catalytic activity	17	9.1892	0.0259851	ENSOCUG00000000786, ENSOCUG00000011082, ENSOCUG00000004691, ENSOCUG00000023796, ENSOCUG00000004067, ENSOCUG00000006405, ENSOCUG00000013357, ENSOCUG00000012306, ENSOCUG00000001230, ENSOCUG00000029153, ENSOCUG00000022921, ENSOCUG00000024304, ENSOCUG00000011610, ENSOCUG00000009566, ENSOCUG00000011786, ENSOCUG00000008779, ENSOCUG00000026988	118	791	9777	1.780721678	36.65634
GOTERM_BP_FAT	GO:0006915~apoptotic process	17	9.1892	0.0638883	ENSOCUG00000000786, ENSOCUG00000008771, ENSOCUG00000016068, ENSOCUG00000017120, ENSOCUG00000009682, ENSOCUG00000006405, ENSOCUG00000000737, ENSOCUG00000014668, ENSOCUG00000001230, ENSOCUG00000000197, ENSOCUG00000006275, ENSOCUG00000024304, ENSOCUG00000011610, ENSOCUG00000009566, ENSOCUG00000029190, ENSOCUG00000011323, ENSOCUG00000004918	118	889	9777	1.584421651	68.17553
GOTERM_BP_FAT	GO:0002682~regulation of immune system process	16	8.6486	0.0312519	ENSOCUG00000008771, ENSOCUG00000016068, ENSOCUG00000023796, ENSOCUG00000016131, ENSOCUG00000013593, ENSOCUG00000017120, ENSOCUG00000002745, ENSOCUG00000004625, ENSOCUG00000007545, ENSOCUG00000000737, ENSOCUG00000007002, ENSOCUG00000006275, ENSOCUG00000024304, ENSOCUG00000011786, ENSOCUG00000013779, ENSOCUG00000008779	118	743	9777	1.784246185	42.34097
GOTERM_BP_FAT	GO:0048646~anatomical structure formation involved in morphogenesis	16	8.6486	0.0453968	ENSOCUG00000000786, ENSOCUG00000011082, ENSOCUG00000008771, ENSOCUG00000016068, ENSOCUG00000016132, ENSOCUG00000005107, ENSOCUG00000010458, ENSOCUG00000013357, ENSOCUG00000015371, ENSOCUG00000000737, ENSOCUG00000011610, ENSOCUG00000007586, ENSOCUG00000011786, ENSOCUG00000006774, ENSOCUG00000026988, ENSOCUG00000009400	118	781	9777	1.69743267	55.32277
GOTERM_BP_FAT	GO:0008544~epidermis development	14	7.5676	6.58E-08	ENSOCUG00000000786, ENSOCUG00000016033, ENSOCUG00000025113, ENSOCUG00000014548, ENSOCUG00000012899, ENSOCUG00000017717, ENSOCUG00000013357, ENSOCUG00000006405, ENSOCUG00000011731, ENSOCUG00000029153, ENSOCUG00000001114, ENSOCUG00000013779, ENSOCUG00000008779, ENSOCUG00000029569	118	163	9777	7.116460435	1.14E-04
GOTERM_BP_FAT	GO:0030855~epithelial cell differentiation	14	7.5676	2.72E-04	ENSOCUG00000000786, ENSOCUG00000005634, ENSOCUG00000025113, ENSOCUG00000014548, ENSOCUG00000012899, ENSOCUG00000012774, ENSOCUG00000006405, ENSOCUG00000011731, ENSOCUG00000001230, ENSOCUG00000001114, ENSOCUG00000029153, ENSOCUG00000013779, ENSOCUG00000008779, ENSOCUG00000029569	118	350	9777	3.314237288	0.470124
GOTERM_BP_FAT	GO:0001816~cytokine production	14	7.5676	9.17E-04	ENSOCUG00000000786, ENSOCUG00000008771, ENSOCUG00000016068, ENSOCUG00000029153, ENSOCUG00000004386, ENSOCUG00000023796, ENSOCUG00000016131, ENSOCUG00000026334, ENSOCUG00000014998, ENSOCUG00000026988, ENSOCUG00000013593, ENSOCUG00000010998, ENSOCUG00000000737, ENSOCUG00000007545	118	398	9777	2.914530279	1.578202
GOTERM_BP_FAT	GO:0043207~response to external biotic stimulus	14	7.5676	0.0029094	ENSOCUG00000000786, ENSOCUG00000016068, ENSOCUG00000023796, ENSOCUG00000013593, ENSOCUG00000017120, ENSOCUG00000002745, ENSOCUG00000023739, ENSOCUG00000007002, ENSOCUG00000008779, ENSOCUG00000015518, ENSOCUG00000029190, ENSOCUG00000010998, ENSOCUG00000022335, ENSOCUG00000022646	118	453	9777	2.560668986	4.927277
GOTERM_BP_FAT	GO:0051707~response to other organism	14	7.5676	0.0029094	ENSOCUG00000000786, ENSOCUG00000016068, ENSOCUG00000023796, ENSOCUG00000013593, ENSOCUG00000017120, ENSOCUG00000002745, ENSOCUG00000023739, ENSOCUG00000007002, ENSOCUG00000008779, ENSOCUG00000015518, ENSOCUG00000029190, ENSOCUG00000010998, ENSOCUG00000022335, ENSOCUG00000022646	118	453	9777	2.560668986	4.927277
GOTERM_BP_FAT	GO:0009607~response to biotic stimulus	14	7.5676	0.0049235	ENSOCUG00000000786, ENSOCUG00000016068, ENSOCUG00000023796, ENSOCUG00000013593, ENSOCUG00000017120, ENSOCUG00000002745, ENSOCUG00000023739, ENSOCUG00000007002, ENSOCUG00000008779, ENSOCUG00000015518, ENSOCUG00000029190, ENSOCUG00000010998, ENSOCUG00000022335, ENSOCUG00000022646	118	482	9777	2.40660384	8.203381
GOTERM_BP_FAT	GO:0098609~cell-cell adhesion	14	7.5676	0.0079395	ENSOCUG00000012863, ENSOCUG00000006498, ENSOCUG00000021242, ENSOCUG00000016131, ENSOCUG00000011842, ENSOCUG00000010458, ENSOCUG00000012103, ENSOCUG00000007545, ENSOCUG00000000737, ENSOCUG00000029153, ENSOCUG00000022921, ENSOCUG00000009566, ENSOCUG00000017373, ENSOCUG00000013779	118	511	9777	2.27002554	12.91079

GOTERM_BP_FAT	GO:0006954~inflammatory response	13	7.027	1.31E-04	ENSOCUG00000000786, ENSOCUG00000008771, ENSOCUG00000016068, ENSOCUG00000016131, ENSOCUG00000004625, ENSOCUG00000007545, ENSOCUG00000002424, ENSOCUG00000009566, ENSOCUG00000011786, ENSOCUG00000014245, ENSOCUG00000014998, ENSOCUG00000026988, ENSOCUG00000022646	118	281	9777	3.833192593	0.226537
GOTERM_BP_FAT	GO:0098542~defense response to other organism	13	7.027	1.54E-04	ENSOCUG00000000786, ENSOCUG00000016068, ENSOCUG00000023796, ENSOCUG00000013593, ENSOCUG00000017120, ENSOCUG00000002745, ENSOCUG00000007002, ENSOCUG00000008779, ENSOCUG00000015518, ENSOCUG00000029190, ENSOCUG00000010998, ENSOCUG00000022335, ENSOCUG00000022646	118	286	9777	3.766178737	0.267313
GOTERM_BP_FAT	GO:0051336~regulation of hydrolase activity	13	7.027	0.0017262	ENSOCUG00000000786, ENSOCUG00000011082, ENSOCUG00000004691, ENSOCUG00000023796, ENSOCUG00000004067, ENSOCUG00000013357, ENSOCUG00000006405, ENSOCUG00000001230, ENSOCUG00000022921, ENSOCUG00000024304, ENSOCUG00000009566, ENSOCUG00000011786, ENSOCUG00000008779	118	375	9777	2.872338983	2.951782
GOTERM_BP_FAT	GO:0043588~skin development	12	6.4865	1.55E-06	ENSOCUG00000000786, ENSOCUG00000016033, ENSOCUG00000029153, ENSOCUG00000001114, ENSOCUG00000014548, ENSOCUG00000012899, ENSOCUG00000006280, ENSOCUG00000013779, ENSOCUG00000029569, ENSOCUG00000006405, ENSOCUG00000013357, ENSOCUG00000011731	118	149	9777	6.672960983	0.002682
GOTERM_BP_FAT	GO:0098602~single organism cell adhesion	12	6.4865	0.0270136	ENSOCUG00000011082, ENSOCUG00000029153, ENSOCUG00000021242, ENSOCUG00000022921, ENSOCUG00000016131, ENSOCUG00000009566, ENSOCUG00000011842, ENSOCUG00000017373, ENSOCUG00000013779, ENSOCUG00000010458, ENSOCUG00000000737, ENSOCUG00000007545	118	476	9777	2.088805014	37.80632
GOTERM_BP_FAT	GO:0009913~epidermal cell differentiation	11	5.9459	2.86E-07	ENSOCUG00000000786, ENSOCUG00000029153, ENSOCUG00000001114, ENSOCUG00000014548, ENSOCUG00000025113, ENSOCUG00000012899, ENSOCUG00000013779, ENSOCUG00000008779, ENSOCUG00000029569, ENSOCUG00000006405, ENSOCUG00000011731	118	100	9777	9.114152542	4.97E-04
GOTERM_BP_FAT	GO:0048871~multicellular organismal homeostasis	11	5.9459	1.48E-04	ENSOCUG00000008771, ENSOCUG00000029153, ENSOCUG00000006275, ENSOCUG00000023796, ENSOCUG00000016131, ENSOCUG00000012899, ENSOCUG00000014245, ENSOCUG00000006405, ENSOCUG00000013357, ENSOCUG00000000737, ENSOCUG00000014668	118	202	9777	4.511956704	0.256728
GOTERM_BP_FAT	GO:0001817~regulation of cytokine production	11	5.9459	0.0117825	ENSOCUG00000000786, ENSOCUG00000008771, ENSOCUG00000016068, ENSOCUG00000029153, ENSOCUG00000023796, ENSOCUG00000016131, ENSOCUG00000026334, ENSOCUG00000014998, ENSOCUG00000013593, ENSOCUG00000010998, ENSOCUG00000000737	118	364	9777	2.503888061	18.57984
GOTERM_BP_FAT	GO:0016337~single organismal cell-cell adhesion	11	5.9459	0.0377992	ENSOCUG00000029153, ENSOCUG00000021242, ENSOCUG00000022921, ENSOCUG00000016131, ENSOCUG00000009566, ENSOCUG00000011842, ENSOCUG00000017373, ENSOCUG00000013779, ENSOCUG00000010458, ENSOCUG00000000737, ENSOCUG00000007545	118	440	9777	2.071398305	48.73844
GOTERM_BP_FAT	GO:0032101~regulation of response to external stimulus	11	5.9459	0.0480407	ENSOCUG00000016068, ENSOCUG00000007002, ENSOCUG00000023796, ENSOCUG00000016131, ENSOCUG00000011610, ENSOCUG00000009566, ENSOCUG00000014245, ENSOCUG00000008779, ENSOCUG00000005107, ENSOCUG00000017120, ENSOCUG00000002745	118	459	9777	1.985654149	57.42083
GOTERM_BP_FAT	GO:0008284~positive regulation of cell proliferation	11	5.9459	0.0534929	ENSOCUG00000011082, ENSOCUG00000016657, ENSOCUG00000005634, ENSOCUG00000029153, ENSOCUG00000008947, ENSOCUG00000023796, ENSOCUG00000011610, ENSOCUG00000011786, ENSOCUG00000029190, ENSOCUG00000011658, ENSOCUG00000000737	118	468	9777	1.947468492	61.45772
GOTERM_BP_FAT	GO:0050673~epithelial cell proliferation	10	5.4054	0.0015259	ENSOCUG00000000786, ENSOCUG00000001230, ENSOCUG00000029153, ENSOCUG00000011610, ENSOCUG00000011786, ENSOCUG00000013779, ENSOCUG00000012774, ENSOCUG00000006405, ENSOCUG00000012306, ENSOCUG00000000737	118	226	9777	3.66619169	2.61345
GOTERM_BP_FAT	GO:0009617~response to bacterium	10	5.4054	0.0025745	ENSOCUG00000023739, ENSOCUG00000000786, ENSOCUG00000016068, ENSOCUG00000023796, ENSOCUG00000015518, ENSOCUG00000008779, ENSOCUG00000013593, ENSOCUG00000029190, ENSOCUG00000002745, ENSOCUG00000022335	118	244	9777	3.395734926	4.372049
GOTERM_BP_FAT	GO:0001568~blood vessel development	10	5.4054	0.0372114	ENSOCUG00000000786, ENSOCUG00000001230, ENSOCUG00000008771, ENSOCUG00000011082, ENSOCUG00000016068, ENSOCUG00000011610, ENSOCUG00000011786, ENSOCUG00000013779, ENSOCUG00000026988, ENSOCUG00000015371	118	378	9777	2.191955878	48.19263
GOTERM_BP_FAT	GO:0001944~vasculature development	10	5.4054	0.0548755	ENSOCUG00000000786, ENSOCUG00000001230, ENSOCUG00000008771, ENSOCUG00000011082, ENSOCUG00000016068, ENSOCUG00000011610, ENSOCUG00000011786, ENSOCUG00000013779, ENSOCUG00000026988, ENSOCUG00000015371	118	407	9777	2.03577229	62.42253
GOTERM_BP_FAT	GO:0030216~keratinocyte differentiation	9	4.8649	8.37E-07	ENSOCUG00000000786, ENSOCUG00000029153, ENSOCUG00000001114, ENSOCUG00000014548, ENSOCUG00000012899, ENSOCUG00000013779, ENSOCUG00000029569, ENSOCUG00000006405, ENSOCUG00000011731	118	64	9777	11.65161547	0.001452
GOTERM_BP_FAT	GO:0042742~defense response to bacterium	9	4.8649	7.17E-05	ENSOCUG00000000786, ENSOCUG00000016068, ENSOCUG00000023796, ENSOCUG00000015518, ENSOCUG00000008779, ENSOCUG00000013593, ENSOCUG00000029190, ENSOCUG00000002745, ENSOCUG00000022335	118	116	9777	6.428477499	0.124233
GOTERM_BP_FAT	GO:0048514~blood vessel morphogenesis	9	4.8649	0.0356249	ENSOCUG00000000786, ENSOCUG00000001230, ENSOCUG00000008771, ENSOCUG00000011082, ENSOCUG00000016068, ENSOCUG00000011610, ENSOCUG00000011786, ENSOCUG00000013779, ENSOCUG00000026988	118	316	9777	2.359820854	46.692
GOTERM_BP_FAT	GO:0031347~regulation of defense response	9	4.8649	0.0658868	ENSOCUG00000016068, ENSOCUG00000007002, ENSOCUG00000023796, ENSOCUG00000016131, ENSOCUG00000009566, ENSOCUG00000014245, ENSOCUG00000008779, ENSOCUG00000017120, ENSOCUG00000002745	118	359	9777	2.077168217	69.33343
GOTERM_BP_FAT	GO:1901342~regulation of vasculature development	8	4.3243	0.0019567	ENSOCUG00000000786, ENSOCUG00000001230, ENSOCUG00000008771, ENSOCUG00000011082, ENSOCUG00000016068, ENSOCUG00000011610, ENSOCUG00000011786, ENSOCUG00000026988	118	148	9777	4.478699038	3.33962

GOTERM_BP_FAT	GO:0050678~regulation of epithelial cell proliferation	8	4.3243	0.0066976	ENSOCUG00000001230, ENSOCUG00000029153, ENSOCUG00000011610, ENSOCUG00000011786, ENSOCUG00000012774, ENSOCUG00000006405, ENSOCUG00000012306, ENSOCUG00000000737	118	185	9777	3.58295923	11.00073
GOTERM_BP_FAT	GO:0043086~negative regulation of catalytic activity	8	4.3243	0.0409916	ENSOCUG00000011082, ENSOCUG00000022921, ENSOCUG00000024304, ENSOCUG00000023796, ENSOCUG00000004067, ENSOCUG00000011786, ENSOCUG00000008779, ENSOCUG00000006405	118	267	9777	2.482574748	51.60923
GOTERM_BP_FAT	GO:0006935~chemotaxis	8	4.3243	0.0856474	ENSOCUG00000000786, ENSOCUG00000029281, ENSOCUG00000016068, ENSOCUG00000011610, ENSOCUG00000009566, ENSOCUG00000002424, ENSOCUG00000002745, ENSOCUG00000000737	118	317	9777	2.091001444	78.83446
GOTERM_BP_FAT	GO:0042330~taxis	8	4.3243	0.0867476	ENSOCUG00000000786, ENSOCUG00000029281, ENSOCUG00000016068, ENSOCUG00000011610, ENSOCUG00000009566, ENSOCUG00000002424, ENSOCUG00000002745, ENSOCUG00000000737	118	318	9777	2.084425967	79.27179
GOTERM_BP_FAT	GO:0045765~regulation of angiogenesis	7	3.7838	0.0059571	ENSOCUG00000000786, ENSOCUG00000008771, ENSOCUG00000011082, ENSOCUG00000016068, ENSOCUG00000011610, ENSOCUG00000011786, ENSOCUG00000026988	118	137	9777	4.233514784	9.842988
GOTERM_BP_FAT	GO:0051346~negative regulation of hydrolase activity	7	3.7838	0.0080605	ENSOCUG00000022921, ENSOCUG00000024304, ENSOCUG00000023796, ENSOCUG00000004067, ENSOCUG00000011786, ENSOCUG00000008779, ENSOCUG00000006405	118	146	9777	3.972544695	13.09489
GOTERM_BP_FAT	GO:0060326~cell chemotaxis	7	3.7838	0.0153732	ENSOCUG00000000786, ENSOCUG00000029281, ENSOCUG00000016068, ENSOCUG00000011610, ENSOCUG00000009566, ENSOCUG00000002424, ENSOCUG00000002745	118	168	9777	3.452330508	23.56083
GOTERM_BP_FAT	GO:0030031~cell projection assembly	7	3.7838	0.0712422	ENSOCUG00000006987, ENSOCUG00000000786, ENSOCUG00000017253, ENSOCUG00000007586, ENSOCUG00000006774, ENSOCUG00000022231, ENSOCUG00000009400	118	243	9777	2.386796401	72.24376
GOTERM_BP_FAT	GO:0001525~angiogenesis	7	3.7838	0.0865199	ENSOCUG00000000786, ENSOCUG00000008771, ENSOCUG00000011082, ENSOCUG00000016068, ENSOCUG00000011610, ENSOCUG00000011786, ENSOCUG00000026988	118	256	9777	2.265591896	79.18198
GOTERM_BP_FAT	GO:0045682~regulation of epidermis development	6	3.2432	2.09E-04	ENSOCUG00000029153, ENSOCUG00000025113, ENSOCUG00000012899, ENSOCUG00000013779, ENSOCUG00000029569, ENSOCUG00000006405	118	46	9777	10.8072955	0.361287
GOTERM_BP_FAT	GO:0045766~positive regulation of angiogenesis	6	3.2432	0.002116	ENSOCUG00000008771, ENSOCUG00000011082, ENSOCUG00000016068, ENSOCUG00000011610, ENSOCUG00000011786, ENSOCUG00000026988	118	76	9777	6.541257806	3.606815
GOTERM_BP_FAT	GO:1904018~positive regulation of vasculature development	6	3.2432	0.0032786	ENSOCUG00000008771, ENSOCUG00000011082, ENSOCUG00000016068, ENSOCUG00000011610, ENSOCUG00000011786, ENSOCUG00000026988	118	84	9777	5.918280872	5.535963
GOTERM_BP_FAT	GO:0052547~regulation of peptidase activity	6	3.2432	0.0209447	ENSOCUG00000001230, ENSOCUG00000024304, ENSOCUG00000011786, ENSOCUG00000009566, ENSOCUG00000008779, ENSOCUG00000006405	118	132	9777	3.766178737	30.72476
GOTERM_BP_FAT	GO:0001818~negative regulation of cytokine production	6	3.2432	0.0228292	ENSOCUG00000000786, ENSOCUG00000029153, ENSOCUG00000016131, ENSOCUG00000026334, ENSOCUG00000013593, ENSOCUG00000010998	118	135	9777	3.682485876	33.00119
GOTERM_BP_FAT	GO:0050878~regulation of body fluid levels	6	3.2432	0.078926	ENSOCUG00000001230, ENSOCUG00000016068, ENSOCUG00000014189, ENSOCUG00000012899, ENSOCUG00000006405, ENSOCUG00000013357	118	191	9777	2.602804153	75.9679
GOTERM_BP_FAT	GO:0042060~wound healing	6	3.2432	0.0993009	ENSOCUG00000008771, ENSOCUG00000016068, ENSOCUG00000014189, ENSOCUG00000005107, ENSOCUG00000013357, ENSOCUG00000025956	118	205	9777	2.425051674	83.69515
GOTERM_BP_FAT	GO:0002526~acute inflammatory response	5	2.7027	0.0031562	ENSOCUG00000008771, ENSOCUG00000016131, ENSOCUG00000002424, ENSOCUG00000004625, ENSOCUG00000007545	118	51	9777	8.123130608	5.334602
GOTERM_BP_FAT	GO:0022404~molting cycle process	5	2.7027	0.0050264	ENSOCUG00000016033, ENSOCUG00000029153, ENSOCUG00000012899, ENSOCUG00000013779, ENSOCUG00000029569	118	58	9777	7.142752776	8.367796
GOTERM_BP_FAT	GO:0001942~hair follicle development	5	2.7027	0.0050264	ENSOCUG00000016033, ENSOCUG00000029153, ENSOCUG00000012899, ENSOCUG00000013779, ENSOCUG00000029569	118	58	9777	7.142752776	8.367796
GOTERM_BP_FAT	GO:0022405~hair cycle process	5	2.7027	0.0050264	ENSOCUG00000016033, ENSOCUG00000029153, ENSOCUG00000012899, ENSOCUG00000013779, ENSOCUG00000029569	118	58	9777	7.142752776	8.367796
GOTERM_BP_FAT	GO:0098773~skin epidermis development	5	2.7027	0.0056726	ENSOCUG00000016033, ENSOCUG00000029153, ENSOCUG00000012899, ENSOCUG00000013779, ENSOCUG00000029569	118	60	9777	6.904661017	9.394416
GOTERM_BP_FAT	GO:0042303~molting cycle	5	2.7027	0.0067423	ENSOCUG00000016033, ENSOCUG00000029153, ENSOCUG00000012899, ENSOCUG00000013779, ENSOCUG00000029569	118	63	9777	6.575867635	11.0701
GOTERM_BP_FAT	GO:0042633~hair cycle	5	2.7027	0.0067423	ENSOCUG00000016033, ENSOCUG00000029153, ENSOCUG00000012899, ENSOCUG00000013779, ENSOCUG00000029569	118	63	9777	6.575867635	11.0701
GOTERM_BP_FAT	GO:0007156~homophilic cell adhesion via plasma membrane adhesion molecules	5	2.7027	0.0079379	ENSOCUG00000012863, ENSOCUG00000006498, ENSOCUG00000029153, ENSOCUG00000010458, ENSOCUG00000012103	118	66	9777	6.276964561	12.90841
GOTERM_BP_FAT	GO:0098742~cell-cell adhesion via plasma-membrane adhesion molecules	5	2.7027	0.028828	ENSOCUG00000012863, ENSOCUG00000006498, ENSOCUG00000029153, ENSOCUG00000010458, ENSOCUG00000012103	118	97	9777	4.27092434	39.78723
GOTERM_BP_FAT	GO:0045861~negative regulation of proteolysis	5	2.7027	0.0297857	ENSOCUG00000016068, ENSOCUG00000024304, ENSOCUG00000011786, ENSOCUG00000008779, ENSOCUG00000006405	118	98	9777	4.22734348	40.80875
GOTERM_BP_FAT	GO:0052548~regulation of endopeptidase activity	5	2.7027	0.0672893	ENSOCUG00000001230, ENSOCUG00000024304, ENSOCUG00000009566, ENSOCUG00000008779, ENSOCUG00000006405	118	128	9777	3.236559852	70.1222
GOTERM_BP_FAT	GO:0031667~response to nutrient levels	5	2.7027	0.0852165	ENSOCUG00000024304, ENSOCUG00000004447, ENSOCUG00000017120, ENSOCUG00000012306, ENSOCUG00000014668	118	139	9777	2.980429216	78.66082
GOTERM_BP_FAT	GO:0031424~keratinization	4	2.1622	5.52E-04	ENSOCUG00000029153, ENSOCUG00000012899, ENSOCUG00000029569, ENSOCUG00000006405	118	14	9777	23.67312349	0.953316
GOTERM_BP_FAT	GO:0045684~positive regulation of epidermis development	4	2.1622	0.0031703	ENSOCUG00000012899, ENSOCUG00000013779, ENSOCUG00000029569, ENSOCUG00000006405	118	25	9777	13.25694915	5.357769
GOTERM_BP_FAT	GO:0010466~negative regulation of peptidase activity	4	2.1622	0.0253659	ENSOCUG00000024304, ENSOCUG00000011786, ENSOCUG00000008779, ENSOCUG00000006405	118	53	9777	6.253277902	35.95428
GOTERM_BP_FAT	GO:0001935~endothelial cell proliferation	4	2.1622	0.0477617	ENSOCUG00000000786, ENSOCUG00000001230, ENSOCUG00000011610, ENSOCUG00000011786	118	68	9777	4.873878365	57.20383
GOTERM_BP_FAT	GO:0009266~response to temperature stimulus	4	2.1622	0.0568193	ENSOCUG00000008771, ENSOCUG00000014245, ENSOCUG00000011842, ENSOCUG00000029190	118	73	9777	4.54005108	63.7405
GOTERM_BP_FAT	GO:0032675~regulation of interleukin-6 production	4	2.1622	0.060655	ENSOCUG00000008771, ENSOCUG00000014998, ENSOCUG00000013593, ENSOCUG00000010998	118	75	9777	4.418983051	66.21449
GOTERM_BP_FAT	GO:0032635~interleukin-6 production	4	2.1622	0.0646091	ENSOCUG00000008771, ENSOCUG00000014998, ENSOCUG00000013593, ENSOCUG00000010998	118	77	9777	4.30420427	68.59783
GOTERM_BP_FAT	GO:0050680~negative regulation of epithelial cell proliferation	4	2.1622	0.0838089	ENSOCUG00000001230, ENSOCUG00000012774, ENSOCUG00000006405, ENSOCUG00000012306	118	86	9777	3.853764289	78.08417

GOTERM_BP_FAT	GO:0043087~regulation of GTPase activity	4	2.1622	0.090695	ENSOCUG00000000786, ENSOCUG00000011082, ENSOCUG00000004691, ENSOCUG00000013357	118	89	9777	3.723862122	80.77187
GOTERM_BP_FAT	GO:0002685~regulation of leukocyte migration	4	2.1622	0.0954133	ENSOCUG00000008771, ENSOCUG00000016068, ENSOCUG00000011786, ENSOCUG00000002745	118	91	9777	3.642018998	82.43071
GOTERM_BP_FAT	GO:2000505~regulation of energy homeostasis	3	1.6216	0.0072774	ENSOCUG00000014245, ENSOCUG00000000737, ENSOCUG00000014668	118	11	9777	22.59707242	11.89729
GOTERM_BP_FAT	GO:0051797~regulation of hair follicle development	3	1.6216	0.0101608	ENSOCUG00000029153, ENSOCUG00000013779, ENSOCUG00000029569	118	13	9777	19.12059974	16.23135
GOTERM_BP_FAT	GO:0050832~defense response to fungus	3	1.6216	0.0101608	ENSOCUG00000023796, ENSOCUG00000010998, ENSOCUG00000022646	118	13	9777	19.12059974	16.23135
GOTERM_BP_FAT	GO:0061436~establishment of skin barrier	3	1.6216	0.0117623	ENSOCUG00000012899, ENSOCUG00000006405, ENSOCUG00000013357	118	14	9777	17.75484262	18.55086
GOTERM_BP_FAT	GO:0033561~regulation of water loss via skin	3	1.6216	0.0134666	ENSOCUG00000012899, ENSOCUG00000006405, ENSOCUG00000013357	118	15	9777	16.57118644	20.95283
GOTERM_BP_FAT	GO:0097009~energy homeostasis	3	1.6216	0.0191705	ENSOCUG00000014245, ENSOCUG00000000737, ENSOCUG00000014668	118	18	9777	13.80932203	28.51516
GOTERM_BP_FAT	GO:0042634~regulation of hair cycle	3	1.6216	0.0191705	ENSOCUG00000029153, ENSOCUG00000013779, ENSOCUG00000029569	118	18	9777	13.80932203	28.51516
GOTERM_BP_FAT	GO:0031069~hair follicle morphogenesis	3	1.6216	0.0212603	ENSOCUG00000016033, ENSOCUG00000012899, ENSOCUG00000029569	118	19	9777	13.08251561	31.11102
GOTERM_BP_FAT	GO:0009620~response to fungus	3	1.6216	0.0234402	ENSOCUG00000023796, ENSOCUG00000010998, ENSOCUG00000022646	118	20	9777	12.42838983	33.72399
GOTERM_BP_FAT	GO:0006953~acute-phase response	3	1.6216	0.0257078	ENSOCUG00000008771, ENSOCUG00000002424, ENSOCUG00000004625	118	21	9777	11.83656174	36.3428
GOTERM_BP_FAT	GO:0050829~defense response to Gram-negative bacterium	3	1.6216	0.0257078	ENSOCUG00000016068, ENSOCUG00000015518, ENSOCUG00000013593	118	21	9777	11.83656174	36.3428
GOTERM_BP_FAT	GO:0048730~epidermis morphogenesis	3	1.6216	0.0257078	ENSOCUG00000016033, ENSOCUG00000012899, ENSOCUG00000029569	118	21	9777	11.83656174	36.3428
GOTERM_BP_FAT	GO:0018149~peptide cross-linking	3	1.6216	0.0257078	ENSOCUG00000025810, ENSOCUG00000001114, ENSOCUG00000014548	118	21	9777	11.83656174	36.3428
GOTERM_BP_FAT	GO:0019730~antimicrobial humoral response	3	1.6216	0.0356082	ENSOCUG00000023796, ENSOCUG00000008779, ENSOCUG00000010998	118	25	9777	9.942711864	46.67597
GOTERM_BP_FAT	GO:0035082~axoneme assembly	3	1.6216	0.0410248	ENSOCUG00000007586, ENSOCUG00000006774, ENSOCUG00000009400	118	27	9777	9.206214689	51.63831
GOTERM_BP_FAT	GO:0050891~multicellular organismal water homeostasis	3	1.6216	0.0467293	ENSOCUG00000012899, ENSOCUG00000006405, ENSOCUG00000013357	118	29	9777	8.571303331	56.39207
GOTERM_BP_FAT	GO:0030104~water homeostasis	3	1.6216	0.05579	ENSOCUG00000012899, ENSOCUG00000006405, ENSOCUG00000013357	118	32	9777	7.767743644	63.04811
GOTERM_BP_FAT	GO:0019217~regulation of fatty acid metabolic process	3	1.6216	0.0721053	ENSOCUG00000017120, ENSOCUG00000006463, ENSOCUG00000000737	118	37	9777	6.718048557	72.68769
GOTERM_BP_FAT	GO:0050906~detection of stimulus involved in sensory perception	3	1.6216	0.0934099	ENSOCUG00000006275, ENSOCUG00000004459, ENSOCUG00000012584	118	43	9777	5.780646433	81.74357
GOTERM_BP_FAT	GO:0001895~retina homeostasis	3	1.6216	0.0971204	ENSOCUG00000029153, ENSOCUG00000006275, ENSOCUG00000023796	118	44	9777	5.649268105	82.99691
GOTERM_BP_FAT	GO:0001578~microtubule bundle formation	3	1.6216	0.0971204	ENSOCUG00000007586, ENSOCUG00000006774, ENSOCUG00000009400	118	44	9777	5.649268105	82.99691
GOTERM_BP_FAT	GO:0019732~antifungal humoral response	2	1.0811	0.0237917	ENSOCUG00000023796, ENSOCUG00000010998	118	2	9777	82.8559322	34.13653
GOTERM_BP_FAT	GO:0090310~negative regulation of methylation-dependent chromatin silencing	2	1.0811	0.0354763	ENSOCUG00000023739, ENSOCUG00000025736	118	3	9777	55.23728814	46.54936
GOTERM_BP_FAT	GO:0045218~zonula adherens maintenance	2	1.0811	0.0354763	ENSOCUG00000022921, ENSOCUG00000017373	118	3	9777	55.23728814	46.54936
GOTERM_BP_FAT	GO:2000507~positive regulation of energy homeostasis	2	1.0811	0.0470221	ENSOCUG00000014245, ENSOCUG00000014668	118	4	9777	41.4279661	56.62377
GOTERM_BP_FAT	GO:0001806~type IV hypersensitivity	2	1.0811	0.0470221	ENSOCUG00000016131, ENSOCUG00000007545	118	4	9777	41.4279661	56.62377
GOTERM_BP_FAT	GO:0090308~regulation of methylation-dependent chromatin silencing	2	1.0811	0.058431	ENSOCUG00000023739, ENSOCUG00000025736	118	5	9777	33.14237288	64.8001
GOTERM_BP_FAT	GO:0031936~negative regulation of chromatin silencing	2	1.0811	0.058431	ENSOCUG00000023739, ENSOCUG00000025736	118	5	9777	33.14237288	64.8001
GOTERM_BP_FAT	GO:0006032~chitin catabolic process	2	1.0811	0.0697043	ENSOCUG00000026988, ENSOCUG00000015772	118	6	9777	27.61864407	71.43582
GOTERM_BP_FAT	GO:0006030~chitin metabolic process	2	1.0811	0.0697043	ENSOCUG00000026988, ENSOCUG00000015772	118	6	9777	27.61864407	71.43582
GOTERM_BP_FAT	GO:0001580~detection of chemical stimulus involved in sensory perception of bitter taste	2	1.0811	0.0697043	ENSOCUG00000006275, ENSOCUG00000012584	118	6	9777	27.61864407	71.43582
GOTERM_BP_FAT	GO:0050912~detection of chemical stimulus involved in sensory perception of taste	2	1.0811	0.0697043	ENSOCUG00000006275, ENSOCUG00000012584	118	6	9777	27.61864407	71.43582
GOTERM_BP_FAT	GO:0045217~cell-cell junction maintenance	2	1.0811	0.0697043	ENSOCUG00000022921, ENSOCUG00000017373	118	6	9777	27.61864407	71.43582
GOTERM_BP_FAT	GO:1901072~glucosamine-containing compound catabolic process	2	1.0811	0.0808439	ENSOCUG00000026988, ENSOCUG00000015772	118	7	9777	23.67312349	76.82111
GOTERM_BP_FAT	GO:0034331~cell junction maintenance	2	1.0811	0.0808439	ENSOCUG00000022921, ENSOCUG00000017373	118	7	9777	23.67312349	76.82111
GOTERM_BP_FAT	GO:0002524~hypersensitivity	2	1.0811	0.0808439	ENSOCUG00000016131, ENSOCUG00000007545	118	7	9777	23.67312349	76.82111
GOTERM_BP_FAT	GO:0006346~methylation-dependent chromatin silencing	2	1.0811	0.0808439	ENSOCUG00000023739, ENSOCUG00000025736	118	7	9777	23.67312349	76.82111
GOTERM_BP_FAT	GO:0046348~amino sugar catabolic process	2	1.0811	0.0808439	ENSOCUG00000026988, ENSOCUG00000015772	118	7	9777	23.67312349	76.82111
GOTERM_CC_FAT	GO:0005576~extracellular region	74	40	2.08E-12	ENSOCUG00000002371, ENSOCUG00000014548, ENSOCUG00000011842, ENSOCUG00000013593, ENSOCUG00000028107, ENSOCUG00000006275, ENSOCUG00000008947, ENSOCUG00000014012, ENSOCUG00000011610, ENSOCUG00000009566, ENSOCUG00000002424, ENSOCUG00000014245, ENSOCUG00000008779, ENSOCUG00000006463, ENSOCUG00000011658, ENSOCUG00000025956, ENSOCUG00000004918, ENSOCUG00000008771, ENSOCUG00000006498, ENSOCUG00000029281, ENSOCUG00000016068, ENSOCUG00000004691, ENSOCUG00000021242, ENSOCUG00000024096, ENSOCUG00000006494, ENSOCUG00000023796, ENSOCUG00000007730, ENSOCUG00000001054, ENSOCUG00000006405, ENSOCUG00000011731, ENSOCUG00000002341, ENSOCUG00000002745, ENSOCUG00000010912, ENSOCUG00000003467, ENSOCUG00000010917, ENSOCUG00000026988, ENSOCUG00000010998, ENSOCUG00000022646, ENSOCUG00000022335, ENSOCUG00000016033, ENSOCUG00000022549, ENSOCUG00000008818, ENSOCUG00000004447, ENSOCUG00000012103, ENSOCUG00000004625, ENSOCUG00000029278, ENSOCUG00000017421, ENSOCUG00000009148, ENSOCUG00000007488, ENSOCUG00000001114, ENSOCUG00000021361, ENSOCUG00000017373, ENSOCUG00000014998, ENSOCUG00000006251, ENSOCUG00000024745, ENSOCUG00000011323, ENSOCUG00000005202, ENSOCUG00000005634, ENSOCUG00000016132, ENSOCUG00000014227, ENSOCUG00000013200, ENSOCUG00000012584, ENSOCUG00000015371, ENSOCUG00000014485, ENSOCUG00000001230, ENSOCUG00000010529, ENSOCUG00000011786, ENSOCUG00000015518, ENSOCUG00000022624, ENSOCUG00000027050, ENSOCUG00000029569, ENSOCUG00000026264, ENSOCUG00000010425, ENSOCUG00000015772	118	2733	8802	2.019721297	2.57E-09

GOTERM_CC_FAT	GO:0044421~extracellular region part	69	37.297	1.05E-11	ENSOCUG00000002371, ENSOCUG00000014548, ENSOCUG00000011842, ENSOCUG00000013593, ENSOCUG00000006275, ENSOCUG00000008947, ENSOCUG00000014012, ENSOCUG00000011610, ENSOCUG00000009566, ENSOCUG00000002424, ENSOCUG00000014245, ENSOCUG00000008779, ENSOCUG00000006463, ENSOCUG00000011658, ENSOCUG00000025956, ENSOCUG00000004918, ENSOCUG00000008771, ENSOCUG00000006498, ENSOCUG00000029281, ENSOCUG00000016068, ENSOCUG00000004691, ENSOCUG00000021242, ENSOCUG00000024096, ENSOCUG00000006494, ENSOCUG00000023796, ENSOCUG00000007730, ENSOCUG00000001054, ENSOCUG00000006405, ENSOCUG00000011731, ENSOCUG00000002341, ENSOCUG00000002745, ENSOCUG00000003467, ENSOCUG00000010917, ENSOCUG00000026988, ENSOCUG00000010998, ENSOCUG00000022335, ENSOCUG00000016033, ENSOCUG00000008818, ENSOCUG00000004447, ENSOCUG00000012103, ENSOCUG00000029278, ENSOCUG00000004625, ENSOCUG00000017421, ENSOCUG00000009148, ENSOCUG00000007488, ENSOCUG00000001114, ENSOCUG00000021361, ENSOCUG00000017373, ENSOCUG00000014998, ENSOCUG00000006251, ENSOCUG00000024745, ENSOCUG00000011323, ENSOCUG00000005202, ENSOCUG00000005634, ENSOCUG00000016132, ENSOCUG00000014227, ENSOCUG00000013200, ENSOCUG00000012584, ENSOCUG00000015371, ENSOCUG00000014485, ENSOCUG00000001230, ENSOCUG00000010529, ENSOCUG00000011786, ENSOCUG00000022624, ENSOCUG00000027050, ENSOCUG00000029569, ENSOCUG00000026264, ENSOCUG00000010425, ENSOCUG00000015772	118	2487	8802	2.069534461	1.29E-08
GOTERM_CC_FAT	GO:0031988~membrane-bounded vesicle	58	31.351	3.78E-08	ENSOCUG00000002371, ENSOCUG00000014548, ENSOCUG00000011842, ENSOCUG00000013593, ENSOCUG00000006275, ENSOCUG00000008947, ENSOCUG00000014012, ENSOCUG00000011610, ENSOCUG00000009566, ENSOCUG00000014245, ENSOCUG00000008779, ENSOCUG00000025956, ENSOCUG00000011658, ENSOCUG00000004918, ENSOCUG00000006498, ENSOCUG00000016068, ENSOCUG00000004691, ENSOCUG00000021242, ENSOCUG00000006494, ENSOCUG00000024096, ENSOCUG00000023796, ENSOCUG00000026334, ENSOCUG00000007730, ENSOCUG00000006405, ENSOCUG00000001054, ENSOCUG00000011731, ENSOCUG00000002341, ENSOCUG00000002745, ENSOCUG00000003467, ENSOCUG00000010917, ENSOCUG00000026988, ENSOCUG00000004852, ENSOCUG00000004067, ENSOCUG00000008818, ENSOCUG00000004447, ENSOCUG00000012103, ENSOCUG00000029278, ENSOCUG00000017421, ENSOCUG00000007488, ENSOCUG00000001114, ENSOCUG00000021361, ENSOCUG00000017373, ENSOCUG00000006251, ENSOCUG00000024745, ENSOCUG00000011323, ENSOCUG00000005202, ENSOCUG00000005634, ENSOCUG00000016132, ENSOCUG00000014227, ENSOCUG00000013200, ENSOCUG00000012584, ENSOCUG00000015371, ENSOCUG00000011786, ENSOCUG00000015518, ENSOCUG00000022624, ENSOCUG00000029569, ENSOCUG00000010425, ENSOCUG00000026264	118	2233	8802	1.937486243	4.68E-05
GOTERM_CC_FAT	GO:0070062~extracellular exosome	53	28.649	2.60E-08	ENSOCUG00000002371, ENSOCUG00000014548, ENSOCUG00000011842, ENSOCUG00000013593, ENSOCUG00000006275, ENSOCUG00000008947, ENSOCUG00000014012, ENSOCUG00000009566, ENSOCUG00000014245, ENSOCUG00000008779, ENSOCUG00000025956, ENSOCUG00000011658, ENSOCUG00000004918, ENSOCUG00000006498, ENSOCUG00000016068, ENSOCUG00000004691, ENSOCUG00000021242, ENSOCUG00000023796, ENSOCUG00000006494, ENSOCUG00000024096, ENSOCUG00000007730, ENSOCUG00000006405, ENSOCUG00000001054, ENSOCUG00000011731, ENSOCUG00000002341, ENSOCUG00000002745, ENSOCUG00000003467, ENSOCUG00000010917, ENSOCUG00000026988, ENSOCUG00000008818, ENSOCUG00000004447, ENSOCUG00000012103, ENSOCUG00000029278, ENSOCUG00000017421, ENSOCUG00000007488, ENSOCUG00000001114, ENSOCUG00000021361, ENSOCUG00000017373, ENSOCUG00000006251, ENSOCUG00000011323, ENSOCUG00000024745, ENSOCUG00000005202, ENSOCUG00000005634, ENSOCUG00000016132, ENSOCUG00000014227, ENSOCUG00000013200, ENSOCUG00000012584, ENSOCUG00000015371, ENSOCUG00000011786, ENSOCUG00000022624, ENSOCUG00000029569, ENSOCUG00000010425, ENSOCUG00000026264	118	1907	8802	2.073120439	3.22E-05
GOTERM_CC_FAT	GO:1903561~extracellular vesicle	53	28.649	3.13E-08	ENSOCUG00000002371, ENSOCUG00000014548, ENSOCUG00000011842, ENSOCUG00000013593, ENSOCUG00000006275, ENSOCUG00000008947, ENSOCUG00000014012, ENSOCUG00000009566, ENSOCUG00000014245, ENSOCUG00000008779, ENSOCUG00000025956, ENSOCUG00000011658, ENSOCUG00000004918, ENSOCUG00000006498, ENSOCUG00000016068, ENSOCUG00000004691, ENSOCUG00000021242, ENSOCUG00000023796, ENSOCUG00000006494, ENSOCUG00000024096, ENSOCUG00000007730, ENSOCUG00000006405, ENSOCUG00000001054, ENSOCUG00000011731, ENSOCUG00000002341, ENSOCUG00000002745, ENSOCUG00000003467, ENSOCUG00000010917, ENSOCUG00000026988, ENSOCUG00000008818, ENSOCUG00000004447, ENSOCUG00000012103, ENSOCUG00000029278, ENSOCUG00000017421, ENSOCUG00000007488, ENSOCUG00000001114, ENSOCUG00000021361, ENSOCUG00000017373, ENSOCUG00000006251, ENSOCUG00000011323, ENSOCUG00000024745, ENSOCUG00000005202, ENSOCUG00000005634, ENSOCUG00000016132, ENSOCUG00000014227, ENSOCUG00000013200, ENSOCUG00000012584, ENSOCUG00000015371, ENSOCUG00000011786, ENSOCUG00000022624, ENSOCUG00000029569, ENSOCUG00000010425, ENSOCUG00000026264	118	1917	8802	2.06230604	3.87E-05

GOTERM_CC_FAT	GO:0043230~extracellular organelle	53	28.649	3.19E-08	ENSOCUG00000002371, ENSOCUG00000014548, ENSOCUG00000011842, ENSOCUG00000013593, ENSOCUG00000006275, ENSOCUG00000008947, ENSOCUG00000014012, ENSOCUG00000009566, ENSOCUG00000014245, ENSOCUG00000008779, ENSOCUG00000025956, ENSOCUG00000011658, ENSOCUG00000004918, ENSOCUG00000006498, ENSOCUG00000016068, ENSOCUG00000004691, ENSOCUG00000021242, ENSOCUG00000023796, ENSOCUG00000006494, ENSOCUG00000024096, ENSOCUG00000007730, ENSOCUG00000006405, ENSOCUG00000001054, ENSOCUG00000011731, ENSOCUG00000002341, ENSOCUG00000002745, ENSOCUG00000003467, ENSOCUG00000010917, ENSOCUG00000026988, ENSOCUG00000008818, ENSOCUG00000004447, ENSOCUG00000012103, ENSOCUG00000029278, ENSOCUG00000017421, ENSOCUG00000007488, ENSOCUG00000001114, ENSOCUG00000021361, ENSOCUG00000017373, ENSOCUG00000006251, ENSOCUG00000011323, ENSOCUG00000024745, ENSOCUG00000005202, ENSOCUG00000005634, ENSOCUG00000016132, ENSOCUG00000014227, ENSOCUG00000013200, ENSOCUG00000012584, ENSOCUG00000015371, ENSOCUG00000011786, ENSOCUG00000022624, ENSOCUG00000029569, ENSOCUG00000010425, ENSOCUG00000026264	118	1918	8802	2.061230802	3.95E-05
GOTERM_CC_FAT	GO:0005615~extracellular space	31	16.757	1.13E-08	ENSOCUG00000008818, ENSOCUG00000013593, ENSOCUG00000004625, ENSOCUG00000029278, ENSOCUG00000009148, ENSOCUG00000006275, ENSOCUG00000014012, ENSOCUG00000011610, ENSOCUG00000009566, ENSOCUG00000002424, ENSOCUG00000014245, ENSOCUG00000014998, ENSOCUG00000006463, ENSOCUG00000029281, ENSOCUG00000008771, ENSOCUG00000005202, ENSOCUG00000016068, ENSOCUG00000023796, ENSOCUG00000011731, ENSOCUG00000014485, ENSOCUG00000015371, ENSOCUG00000012584, ENSOCUG00000001230, ENSOCUG00000010529, ENSOCUG00000011786, ENSOCUG00000026988, ENSOCUG00000027050, ENSOCUG00000026264, ENSOCUG00000015772, ENSOCUG00000010998, ENSOCUG00000022335	118	724	8802	3.193908606	1.40E-05
GOTERM_CC_FAT	GO:0030054~cell junction	17	9.1892	0.0089411	ENSOCUG00000000786, ENSOCUG00000006498, ENSOCUG00000021242, ENSOCUG00000003831, ENSOCUG00000010458, ENSOCUG00000012103, ENSOCUG00000004459, ENSOCUG00000014485, ENSOCUG00000002745, ENSOCUG00000010156, ENSOCUG00000029153, ENSOCUG00000007002, ENSOCUG00000022921, ENSOCUG00000021361, ENSOCUG00000017373, ENSOCUG00000025956, ENSOCUG00000004918	118	630	8802	2.01283293	10.5269
GOTERM_CC_FAT	GO:0045111~intermediate filament cytoskeleton	10	5.4054	2.89E-05	ENSOCUG00000017421, ENSOCUG00000013920, ENSOCUG00000016132, ENSOCUG00000014548, ENSOCUG00000021297, ENSOCUG00000017694, ENSOCUG00000012774, ENSOCUG00000029569, ENSOCUG00000011731, ENSOCUG00000029278	118	120	8802	6.216101695	0.035821
GOTERM_CC_FAT	GO:0070161~anchoring junction	10	5.4054	0.0283404	ENSOCUG00000000786, ENSOCUG00000029153, ENSOCUG00000021242, ENSOCUG00000022921, ENSOCUG00000021361, ENSOCUG00000017373, ENSOCUG00000010458, ENSOCUG00000012103, ENSOCUG00000025956, ENSOCUG00000004918	118	324	8802	2.302259887	29.95717
GOTERM_CC_FAT	GO:0005911~cell-cell junction	9	4.8649	0.0188958	ENSOCUG00000006498, ENSOCUG00000010156, ENSOCUG00000029153, ENSOCUG00000021242, ENSOCUG00000007002, ENSOCUG00000022921, ENSOCUG00000017373, ENSOCUG00000010458, ENSOCUG00000012103	118	252	8802	2.664043584	21.0426
GOTERM_CC_FAT	GO:0005912~adherens junction	9	4.8649	0.0557547	ENSOCUG00000000786, ENSOCUG00000029153, ENSOCUG00000022921, ENSOCUG00000021361, ENSOCUG00000017373, ENSOCUG00000010458, ENSOCUG00000012103, ENSOCUG00000025956, ENSOCUG00000004918	118	312	8802	2.15172751	50.8608
GOTERM_CC_FAT	GO:0005882~intermediate filament (keratins)	8	4.3243	1.49E-04	ENSOCUG00000017421, ENSOCUG00000013920, ENSOCUG00000016132, ENSOCUG00000017694, ENSOCUG00000012774, ENSOCUG00000029569, ENSOCUG00000011731, ENSOCUG00000029278	118	87	8802	6.859146698	0.184079
GOTERM_CC_FAT	GO:0005913~cell-cell adherens junction	5	2.7027	6.22E-04	ENSOCUG00000029153, ENSOCUG00000022921, ENSOCUG00000017373, ENSOCUG00000010458, ENSOCUG00000012103	118	30	8802	12.43220339	0.767871
GOTERM_CC_FAT	GO:0045095~keratin filament	4	2.1622	0.0085753	ENSOCUG00000017421, ENSOCUG00000013920, ENSOCUG00000012774, ENSOCUG00000029278	118	32	8802	9.324152542	10.11708
GOTERM_CC_FAT	GO:0044447~axoneme part	3	1.6216	0.008909	ENSOCUG00000010057, ENSOCUG00000007586, ENSOCUG00000009400	118	11	8802	20.34360555	10.49111
GOTERM_CC_FAT	GO:0030286~dynein complex	3	1.6216	0.0529051	ENSOCUG00000006833, ENSOCUG00000010057, ENSOCUG00000009400	118	28	8802	7.992130751	48.99229
GOTERM_CC_FAT	GO:0005858~axonemal dynein complex	2	1.0811	0.077172	ENSOCUG00000010057, ENSOCUG00000009400	118	6	8802	24.86440678	63.01514
GOTERM_CC_FAT	GO:0005915~zonula adherens	2	1.0811	0.077172	ENSOCUG00000022921, ENSOCUG00000017373	118	6	8802	24.86440678	63.01514
GOTERM_CC_FAT	GO:0001533~cornified envelope	2	1.0811	0.089447	ENSOCUG00000014548, ENSOCUG00000006494	118	7	8802	21.31234867	68.6672
GOTERM_MF_FAT	GO:0005509~calcium ion binding	13	7.027	0.0026924	ENSOCUG00000012863, ENSOCUG00000006498, ENSOCUG00000005202, ENSOCUG00000014189, ENSOCUG00000024096, ENSOCUG00000011842, ENSOCUG00000012103, ENSOCUG00000002745, ENSOCUG00000022645, ENSOCUG00000029153, ENSOCUG00000001114, ENSOCUG00000009566, ENSOCUG00000022646	106	449	9935	2.713682397	3.533253
GOTERM_MF_FAT	GO:0005198~structural molecule activity	12	6.4865	0.0027963	ENSOCUG00000017421, ENSOCUG00000013920, ENSOCUG00000007002, ENSOCUG00000016132, ENSOCUG00000014548, ENSOCUG00000017694, ENSOCUG00000027112, ENSOCUG00000012774, ENSOCUG00000029569, ENSOCUG00000011731, ENSOCUG00000014485, ENSOCUG00000029278	106	392	9935	2.869175972	3.667289
GOTERM_MF_FAT	GO:0098772~molecular function regulator	12	6.4865	0.084792	ENSOCUG00000022645, ENSOCUG00000016068, ENSOCUG00000017253, ENSOCUG00000024304, ENSOCUG00000023796, ENSOCUG00000014012, ENSOCUG00000011610, ENSOCUG00000023409, ENSOCUG00000008779, ENSOCUG00000012306, ENSOCUG00000015371, ENSOCUG00000006463	106	655	9935	1.717125162	69.33971
GOTERM_MF_FAT	GO:0004857~enzyme inhibitor activity	6	3.2432	0.0318169	ENSOCUG00000016068, ENSOCUG00000014012, ENSOCUG00000008779, ENSOCUG00000012306, ENSOCUG00000015371, ENSOCUG00000006463	106	167	9935	3.367416111	35.04128
GOTERM_MF_FAT	GO:0001228~transcriptional activator activity, RNA polymerase II transcription regulatory region sequence-specific binding	6	3.2432	0.0809425	ENSOCUG00000005775, ENSOCUG00000004386, ENSOCUG00000017717, ENSOCUG00000015184, ENSOCUG00000013357, ENSOCUG00000000737	106	218	9935	2.579626104	67.57365

GOTERM_MF_FAT	GO:0005506~iron ion binding	5	2.7027	0.0701013	ENSOCUG00000023380, ENSOCUG00000001230, ENSOCUG00000011201, ENSOCUG00000023796, ENSOCUG00000004655	106	147	9935	3.187973303	62.08151
GOTERM_MF_FAT	GO:0001077~transcriptional activator activity, RNA polymerase II core promoter proximal region sequence-specific binding	5	2.7027	0.0846018	ENSOCUG00000005775, ENSOCUG00000004386, ENSOCUG00000017717, ENSOCUG00000015184, ENSOCUG00000000737	106	157	9935	2.984917678	69.25459
GOTERM_MF_FAT	GO:0004866~endopeptidase inhibitor activity	4	2.1622	0.066104	ENSOCUG00000016068, ENSOCUG00000014012, ENSOCUG00000008779, ENSOCUG00000015371	106	88	9935	4.260291595	59.84804
GOTERM_MF_FAT	GO:0030414~peptidase inhibitor activity	4	2.1622	0.0697277	ENSOCUG00000016068, ENSOCUG00000014012, ENSOCUG00000008779, ENSOCUG00000015371	106	90	9935	4.165618449	61.8777
GOTERM_MF_FAT	GO:0061135~endopeptidase regulator activity	4	2.1622	0.0734398	ENSOCUG00000016068, ENSOCUG00000014012, ENSOCUG00000008779, ENSOCUG00000015371	106	92	9935	4.075061526	63.85816
GOTERM_MF_FAT	GO:0005003~ephrin receptor activity	3	1.6216	0.0068148	ENSOCUG00000000786, ENSOCUG00000011082, ENSOCUG00000007545	106	12	9935	23.43160377	8.720015
GOTERM_MF_FAT	GO:0031490~chromatin DNA binding	3	1.6216	0.0498542	ENSOCUG00000017717, ENSOCUG00000013357, ENSOCUG00000014668	106	34	9935	8.269977802	49.45644
GOTERM_MF_FAT	GO:0016712~oxidoreductase activity, acting on paired donors, with incorporation or reduction of molecular oxygen, reduced flavin or flavoprotein as one donor, and incorporation of one atom of oxygen	3	1.6216	0.0498542	ENSOCUG00000023380, ENSOCUG00000011201, ENSOCUG00000004655	106	34	9935	8.269977802	49.45644
GOTERM_MF_FAT	GO:0004867~serine-type endopeptidase inhibitor activity	3	1.6216	0.0695138	ENSOCUG00000016068, ENSOCUG00000008779, ENSOCUG00000015371	106	41	9935	6.858030373	61.76057
GOTERM_MF_FAT	GO:0004714~transmembrane receptor protein tyrosine kinase activity	3	1.6216	0.0755294	ENSOCUG00000000786, ENSOCUG00000011082, ENSOCUG00000007545	106	43	9935	6.539052216	64.93066
GOTERM_MF_FAT	GO:0008061~chitin binding	2	1.0811	0.0416155	ENSOCUG00000026988, ENSOCUG00000015772	106	4	9935	46.86320755	43.28554
GOTERM_MF_FAT	GO:0004568~chitinase activity	2	1.0811	0.0617753	ENSOCUG00000026988, ENSOCUG00000015772	106	6	9935	31.24213836	57.29258
GOTERM_MF_FAT	GO:0003810~protein-glutamine gamma-glutamyltransferase activity	2	1.0811	0.0716971	ENSOCUG00000025810, ENSOCUG00000001114	106	7	9935	26.77897574	62.94057
KEGG_PATHWAY	ocu04514:Cell adhesion molecules (CAMs)	4	2.1622	0.0730994	ENSOCUG00000029153, ENSOCUG00000007002, ENSOCUG00000010458, ENSOCUG00000025956	51	143	7342	4.026875086	58.48335
KEGG_PATHWAY	ocu04115:p53 signaling pathway	3	1.6216	0.08804	ENSOCUG00000016068, ENSOCUG00000006405, ENSOCUG00000004238	51	73	7342	5.916196616	65.60516
